# Novel tau fragments in cerebrospinal fluid: relation to tangle pathology and cognitive decline in Alzheimer’s disease

**DOI:** 10.1007/s00401-018-1948-2

**Published:** 2018-12-13

**Authors:** Claudia Cicognola, Gunnar Brinkmalm, Jessica Wahlgren, Erik Portelius, Johan Gobom, Nicholas C. Cullen, Oskar Hansson, Lucilla Parnetti, Radu Constantinescu, Kristin Wildsmith, Hsu-Hsin Chen, Thomas G. Beach, Tammaryn Lashley, Henrik Zetterberg, Kaj Blennow, Kina Höglund

**Affiliations:** 1 Institute of Neuroscience and Physiology, Department of Psychiatry and Neurochemistry, The Sahlgrenska Academy at University of Gothenburg, Göteborgsvägen 31, House V3/SU, 43180 Mölndal, Sweden; 2 Clinical Neurochemistry Laboratory, Sahlgrenska University Hospital, Mölndal, Sweden; 3 Department of Neurology, Perelman School of Medicine, University of Pennsylvania, Philadelphia, PA USA; 4 Clinical Memory Research Unit, Department of Clinical Sciences Malmö, Lund University, Lund, Sweden; 5 Memory Clinic, Skåne University Hospital, Malmö, Sweden; 6 Department of Medicine, Center for Memory Disturbances, Laboratory of Clinical Neurochemistry, Neurology Clinic, University of Perugia, Santa Maria della Misericordia Hospital, Perugia, Italy; 7 Institute of Neuroscience and Physiology, Department of Neurology, The Sahlgrenska Academy at University of Gothenburg, Gothenburg, Sweden; 8 Biomarker Development Department, Genentech, South San Francisco, CA USA; 9 Biomarker Discovery Department, Genentech, South San Francisco, CA USA; 10 Civin Laboratory for Neuropathology, Banner Sun Health Research Institute, Sun City, AZ USA; 11 Queen Square Brain Bank for Neurological Disorders, Department of Movement Disorders, Institute of Neurology, University College London, London, UK; 12 Department of Neurodegenerative Disease, UCL Institute of Neurology Queen Square, London, UK; 13 UK Dementia Research Institute at UCL, London, UK; 14 Department of Neurobiology, Care Sciences and Society, Center for Alzheimer Research, Division for Neurogeriatrics, Karolinska Institutet, Novum, Huddinge, Stockholm, Sweden

**Keywords:** Alzheimer’s disease, Tau fragments, Cerebrospinal fluid, Mass spectrometry, Immunohistochemistry

## Abstract

Tau is an axonal microtubule-binding protein. Tau pathology in brain and increased tau concentration in the cerebrospinal fluid (CSF) are hallmarks of Alzheimer’s disease (AD). Most of tau in CSF is present as fragments. We immunoprecipitated tau from CSF and identified several endogenous peptides ending at amino acid (aa) 123 or 224 using high-resolution mass spectrometry. We raised neo-epitope-specific antibodies against tau fragments specifically ending at aa 123 and 224, respectively. With these antibodies, we performed immunohistochemistry on brain tissue and designed immunoassays measuring N-123, N-224, and x-224 tau. Immunoassays were applied to soluble brain fractions from pathologically confirmed subjects (81 AD patients, 33 controls), CSF from three cross-sectional and two longitudinal cohorts (a total of 133 AD, 38 MCI, 20 MCI-AD, 31 PSP, 15 CBS patients, and 91 controls), and neuronally- and peripherally-derived extracellular vesicles (NDEVs and PDEVs, respectively) in serum from four AD patients and four controls. Anti-tau 224 antibody stained neurofibrillary tangles and neuropil threads, while anti-tau 123 only showed weak cytoplasmic staining in AD. N-224 tau was lower in the AD soluble brain fraction compared to controls, while N-123 tau showed similar levels. N-224 tau was higher in AD compared to controls in all CSF cohorts (*p* < 0.001), but not N-123 tau. Decrease in cognitive performance and conversion from MCI to AD were associated with increased baseline CSF levels of N-224 tau (*p* < 0.0001). N-224 tau concentrations in PSP and CBS were significantly lower than in AD (*p* < 0.0001) and did not correlate to t-tau and p-tau. In a longitudinal cohort, CSF N-224 tau levels were stable over 6 months, with no significant effect of treatment with AChE inhibitors. N-224 tau was present in NDEVs, while N-123 tau showed comparable concentrations in both vesicle types. We suggest that N-123 tau is produced both in CNS and PNS and represents a general marker of tau metabolism, while N-224 tau is neuron-specific, present in the tangles, secreted in CSF, and upregulated in AD, suggesting a link between tau cleavage and propagation, tangle pathology, and cognitive decline.

**Electronic supplementary material:**

The online version of this article (10.1007/s00401-018-1948-2) contains supplementary material, which is available to authorized users.

## Introduction

Alzheimer’s disease (AD) is the most common neurodegenerative tauopathy and is responsible for 75% of the 35.6 million dementia cases worldwide [44]. As the world population ages, the frequency is expected to double by 2030 and triple by 2050 [44]. At present, the only therapeutic option is symptomatic, while disease-modifying therapies are still under development [37].

The classical pathological hallmarks of AD consist of deposition of amyloid beta (Aβ) in cortical plaques and hyperphosphorylation of tau and formation of neurofibrillary tangles (NFT) causing neuronal degeneration [37]. In cerebrospinal fluid (CSF), these pathological changes are reflected by low Aβ42 levels, low Aβ42/Aβ40 ratio, and high levels of phosphorylated tau (p-tau) and total tau (t-tau) [12, 32]. Tau pathology, namely the aggregation of tau and formation of tangles, is described as a mechanism common to AD and primary tauopathies, such as corticobasal neurodegeneration (CBD) and progressive supranuclear palsy (PSP). Tau pathology is predominantly localized to neurons in AD, while in primary tauopathies is also present in the glia in filamentous (tufted astrocytes, in PSP) or more diffuse form (astrocytic plaques, in CBD). Their clinical presentation is very different, with AD being primarily characterized by memory impairment and PSP by parkinsonism; CBD has a very heterogeneous clinical presentation, ranging from parkinsonism to AD-like symptoms, which goes under the name of corticobasal syndrome (CBS) [7]. The use of AD biomarkers to better differentiate AD-like CBS has been proposed to help diversify the therapeutic options [15]. Nonetheless, several studies on CSF in primary tauopathies show normal or even low concentrations of t-tau and p-tau, in stark contrast to the increased levels seen in AD, and no CSF profile specific for primary tauopathies has been established [15, 21, 41, 42].

**Fig. 1:**
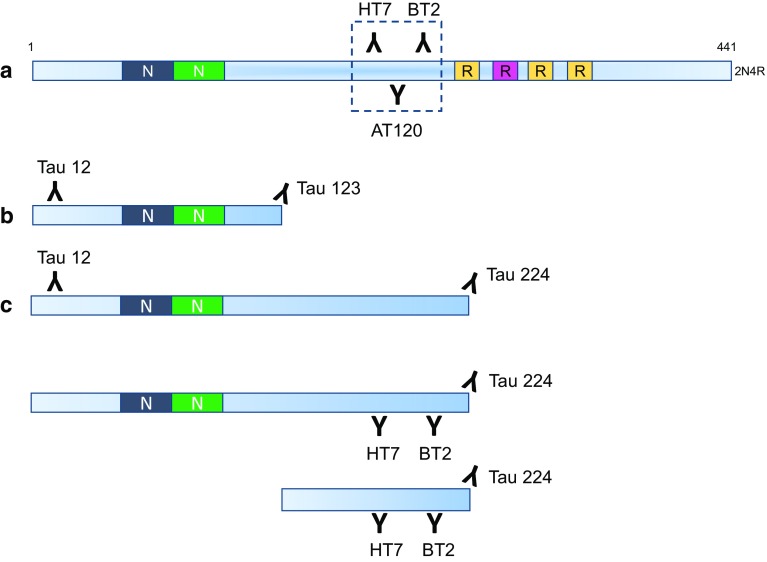
Overview of standard (**a**) and novel (**b**, **c**) immunoassays for t-tau and tau fragments: the traditional assay only detects the mid-region of tau; our novel immunoassays detect fragments ending at aa123 and aa224

From a methodological perspective, most of the data on CSF t-tau come from studies based on commercially available immunoassays that rely on a combination of monoclonal antibodies (AT120, HT7, and BT2) having epitopes in the mid-region of the protein (Fig. 1a), but several studies suggest that tau is present as different fragments in both brain extract and CSF [3, 9, 10, 22, 30, 35, 38]. A recent study used high-performance liquid chromatography (HPLC) separation of CSF proteins, followed by Western blotting with tau antibodies binding to different tau domains, to show that CSF tau is composed of a series of fragments, with N-terminal and mid-region tau representing the most abundant variants in CSF [30]. Mass spectrometry (MS) has also proven helpful to characterize tau species in detail. However, CSF is a complex matrix and several pre-treatments had to be tested to reach the adequate sensitivity to detect tau and its variants in CSF [9, 10, 35]. Different groups have detected tau peptides spanning from the N- to the C-terminus of the protein in CSF [9, 10, 35]. However, the most reproducible result so far is increased concentrations of C-terminally truncated tau forms in AD, which fits well with the highly reproducible results that have been generated using regular t-tau and p-tau immunoassays [32], whilst the other neurodegenerative dementias, including primary tauopathies, are surprisingly normal [9, 10].

A possible explanation for the AD specificity of CSF tau profiles has been proposed, based on an in vivo model using stable-isotope labeling on human central nervous system (CNS) and iPSC-derived neurons [36]. The results show that most tau in CSF and cell media lack the microtubule-binding region and more C-terminal parts, that these tau forms are actively secreted from neurons, and that the secretion appears to be stimulated by Aβ exposure [36]. CSF tau concentrations generated using the currently available assays would thus not be specific for tangle pathology or neurodegeneration but rather reflect a neuronal response to Aβ pathology.

To identify novel tau biomarkers that might better reflect AD-type neurodegeneration, we used N-terminal and mid-region-specific antibodies for enrichment of tau species followed by MS analysis using both bottom–up and top–down approaches. We identified a large number of endogenous and tryptic peptides belonging to two different pools of tau, one starting at the N-terminal and ending at aa 123 through 129 and another mid-region pool covering aa 144–224 (tau 441 numbering). We then immunized mice to generate neo-epitope-specific antibodies against aa 123 and 224. We applied these unique antibodies for further studies evaluating the link between these specific tau fragments and tau pathology. We immunohistochemically stained post-mortem brain and developed immunoassays to specifically quantify the respective fragment in soluble brain extracts and CSF from AD, PSP, CBS patients, and controls. We also evaluated the N-224 fragment stability as biomarker in a longitudinal cohort under treatment with acetylcholinesterase (AChE) inhibitors. Furthermore, we analyzed the two fragments in neuronally derived extracellular vesicles (NDEV), including exosomes, isolated from serum, to study the potential role of exosomes in tau propagation. The present study aimed to characterize new tau fragments and describe a possible model for their distribution, function, and role in the pathogenesis of AD.

## Materials and methods

### Brain tissue extraction for immunoprecipitation

Brain tissue for the immunoprecipitation mass spectrometry (IP-MS) analysis was from the Netherlands Brain Bank. Samples were collected from the superior parietal lobe of control brains (Braak 0–I) 4–7 h post-mortem and stored at − 80 °C. Brain material was kept on dry ice and 100 mg samples were excised and put in TBS solution (20 mM Tris, 137 mM NaCl, and pH 7.6) containing protease inhibitor (Protease Inhibitor Cocktail tablets, Roche, Cat no: 11 697 498 001, Lot nr: 14,251,300) and homogenized with a motor homogenizer to final a tissue:TBS ratio of 1:5. The tissue homogenates were further diluted 1:2 in TBS solution with protease inhibitor and vortexed. The samples were then centrifuged for 60 min at 31,000×*g*, + 4 °C. Supernatants were collected, aliquoted, and stored at − 80 °C pending analysis. Aliquots were further diluted 1:40 in TBS prior to IP.

### Immunoprecipitation

Aliquots (4 µg) of Tau 12 (binding region aa 9–18, Nordic Biosite), HT7 (aa 159–163, Thermo Scientific), and BT2 (aa 194–198, Thermo Scientific) were added to 50 µL/each magnetic Dynabeads M-280 and incubated 2 h on a rocking platform at room temperature. The beads were washed three times with double volume of PBS (10 mM Na-phosphate, 0.15 M NaCl, and pH 7.4). The antibody was cross-linked using 20 mM dimethyl pimelimidate dihydrochloride (DMP; Sigma-Aldrich) and 0.2 M triethanolamine (pH 8.2, Sigma-Aldrich) according to the manufacturer’s product description. The cross-linked beads were washed twice in PBS and blocked with Roti-Block (Carl Roth) for 1 h on a rocking platform at room temperature. Antibody-conjugated beads and Tween 20 (final concentration in the sample: 0.025%) were added to 3 mL CSF or 7 µL of a TBS-soluble fraction of brain homogenate diluted in 270 µL PBS. For some of the samples intended for tryptic digestion, 100 fmol of a 13C15N lysine- and arginine-labeled tau 1N4R protein standard (provided by Dr. Thomas McAvoy, Merck Research Laboratories) were added. Samples were incubated overnight on a rocking platform at + 4 °C. The magnetic beads/sample solution was transferred to a magnetic particle processor (KingFisher, Thermo Fisher Scientific) (tube 1). The following three wash steps (tubes 2–4) were conducted for 10 s in 1 mL of each washing buffer: 0.025% Tween 20 in PBS (tube 2), PBS (tube3), and 50 mM ammonium hydrogen carbonate (tube 4, pH 8.0). Tau was eluted from the beads by adding 100 µL 0.5% formic acid (tube 5) for 4 min. The eluted fractions were transferred to 0.65 mL prelubricated microcentrifuge tubes (Costar, Cat. 3206) and dried in a vacuum centrifuge. After drying, 10 µL of a solution of 1 µg trypsin in 390 µL 50 mM ammonium hydrogen carbonate was added to half of the samples and incubated overnight at + 37 °C. The reaction was stopped by adding 2 µL of 10% formic acid in ultra-pure water. The other half was analyzed intact for endogenous peptides. Samples were dried in a vacuum centrifuge and stored at − 80 °C pending analysis. Following the same protocol described above, we immunoprecipitated tau from CSF and brain lysate with the neo-epitope-specific antibodies to verify their specificity.

### LC–MS/MS analysis

NanoLC coupled to ESI high-resolution hybrid quadrupole-orbitrap MS was performed with a Dionex 3000 system and either a Q Exactive or an Orbitrap Fusion (all three from Thermo Fisher Scientific, Inc.) in a similar way as previously published [15]. Immunoprecipitated samples were reconstituted in 7 µL 8% formic acid/8% acetonitrile in water. Samples (6 µl) were loaded onto an Acclaim PepMap C18 trap column (length 20 mm, internal diameter 75 µm, particle size 3 µm, pore size 100 Å, Thermo Fisher Scientific, Inc.) for desalting and sample clean-up. Sample loading buffer was 0.05% trifluoroacetic acid in water. Separation was performed by reversed-phase Acclaim PepMap C18 analytical columns (lengths 150 or 500 mm, internal diameter 75 µm, particle size 2 µm, pore size 100 Å, and Thermo Fisher Scientific, Inc.). Separation was performed at a flow rate of 300 or 150 nL/min by applying a 50-min-long linear gradient from 3 to 40% B. Buffer A was 0.1% formic acid in water and buffer B was 0.1% formic acid/84% acetonitrile in water. The mass spectrometer was set to operate in data-dependent mode using higher energy collision-induced dissociation (HCD) for ion fragmentation. For endogenous peptides, the acquisition parameters were optimized for large fragments. Acquisition settings were the same for MS and MS/MS acquisitions for a better quality MS/MS data: resolution setting 70,000, 1 microscan, target values 106, and trap injection time 250 ms. For trypsinated samples, the MS/MS resolution setting was 17,500 and the trap injection time was 60 ms.

### Processing of LC–MS/MS data

LC–MS/MS acquisitions of tryptic digests were processed directly using Proteome Discoverer v1.4 (Thermo Fisher Scientific, Inc.) and Mascot database search software v2.6.1 (Matrix Science). Searches were made against both Uniprot and a custom made tau-only database. Acquisitions of non-digested samples were processed using Mascot Daemon v2.6/Mascot Distiller v2.6.3 (both Matrix Science) for charge and isotope deconvolution before submitting searches using Mascot. The same databases as for the digested samples were utilized. Processing and search settings are described in [17] and supplementary information.

### Expression of recombinant Tau 123 and 224

Tau 123 (aa 1–123 of Tau F) and Tau 224 (aa 1–224) were PCR amplified using primers containing 5′ Sgf I and 3′ Not I sites with cDNA for Tau F (RC213312, Origene) as template. The PCR fragments were purified and cloned into Sgf I/Not I digested pEX_N_His_GST expression plasmid (PS100028, OriGene Technologies). 5′ primer sequence was TTCTAAGCGATCGCCATGGCTGAGCCCC, 3′ primer sequence for the 123 fragment was TAAAGCGGCCGCTTAGGTCACGTGACCAGCAGCTT, and 3′ primer sequence for the 224 fragment was TAAAGCGGCCGCTTACTTGGGCTCCCGGGTGGGT. Constructs were sequenced and transfected into E. coli BL21 (DE3). E. coli BL21 (DE3) containing the different constructs was incubated overnight in 50 mL LB medium with ampicillin at a concentration of 100 µg/mL. The overnight culture was used to inoculate 1 L of LB media with ampicillin (100 µg/mL), and when OD600 reached 0.4–0.6, protein expression was induced with 0.5 mM IPTG for 4 h at + 37 °C. The culture was centrifuged at 5500×*g* for 20 min at + 4 °C and the dry weight was calculated. The pellet was stored at − 20 °C pending purification. The pellet was resuspended in 5 mL/mg of lysis buffer (20 mM Tris, 150 mM NaCl, and 1% NP40 pH 7.5) plus complete protease inhibitor (Roche) and incubated with rotation for 30 min at room temperature, after which the lysate was centrifuged at 17,000×*g* for 20 min at + 4 °C and the supernatant was collected. Protein extract was added to 50% Glutathione-Sepharose 4b (GE Healthcare) equilibrated with PBS and incubated for 30 min with rotation at room temperature. Sepharose was washed with PBS and the GST-Tau fusion protein was incubated with elution buffer (100 mM Tris–HCl and 120 mM NaCl pH 8.0, with 20 mM glutathione) for 10 min. The unbound fusion protein was eluted and the incubation step was repeated four times. Thrombin cleavage of the GST-fusion protein bound to the GST Sepharose was done in PBS with 50 U thrombin for two and a half hours at room temperature. The cleaved, untagged protein was eluted with PBS containing protease inhibitor.

### Generation of monoclonal antibodies

Monoclonal antibodies against the recombinant protein fragments were generated by immunization of 8-week-old Balb/c mice with KLH-conjugated peptides (Caslo) in complete Freund’s adjuvant (Sigma). Peptide sequence was (KLH)-CEEAGIGDTPSLEDEAAGHVT for the 123 and (KLH)-CGGGRTPSLPTPPTREPK for the 224. After 2–3 dosages with the recombinant protein fragment (approximately 75 µg/mouse), the spleen was removed and B cells were fused with the myeloma cell line SP2/0 following the standard procedures. Approximately 10 days after fusion, cell media were screened for the antibodies using full-length recombinant tau and recombinant protein fragments. Clones that reacted with the recombinant protein fragments but not with full-length tau and negative control protein were further grown, subcloned, and subsequently frozen in liquid nitrogen. The isotype was determined using a commercially available kit (Pierce Rapid Isotyping Kit-Mouse). Finally, antibodies were purified using a protein G column (GE healthcare).

### Immunohistochemistry

Immunohistochemical analysis of the tau antibodies was carried out on brains that were donated to the Queen Square Brain Bank for Neurological Disorders, UCL Institute of Neurology, University College London. Seven-micron-thick formalin-fixed paraffin-embedded tissue sections were cut from the frontal cortex of pathologically diagnosed AD cases and neurologically normal controls. Tau immunohistochemistry required pressure cooker pre-treatment in citrate buffer pH 6.0. Endogenous peroxidase activity was blocked with 0.3% H202 in methanol and non-specific binding with 10% dried milk solution. Tissue sections were incubated with the primary anti-tau 123, anti-tau 224, and AT8 (binding to Ser202 and Thr205) antibodies for 1 h at room temperature, followed by biotinylated anti-mouse IgG (1:200, 30 min; DAKO) and ABC complex (30 min; DAKO). Colour was developed with di-aminobenzidine/H_2_0_2_ [26]. Sections were viewed and imaged on a Nikon Eclipse.

### ELISA assay for Tau N-123

The immunoassay N-123, detecting fragments going from the N-terminal (antibody-binding region: aa 9–18) to aa 123, was developed on an ELISA platform (Fig. 1b). 96-well plates were coated and incubated overnight at + 4 °C with in house antibody anti-tau 123 at a concentration of 6 µg/mL in carbonate buffer (pH 9.6). After blocking and washing, 90 µL of titrated calibrator (123 recombinant tau fragment) and sample were co-incubated with 10 µL of biotinylated detection antibody Tau 12 (Nordic Biosite), at a final concentration of 0.617 µg/mL. CSF samples were run neat, brain tissue extracts were run at 50-fold dilution. After overnight incubation at + 4 °C, the plate was washed and incubated for 30 min with a streptavidin-HRP-conjugated solution (KemEnTek Diagnostics). After a final wash and addition of the chromogen tetramethylbenzidine (TMB ONE, KemEnTek Diagnostics), the plate was read on a Magellan reader at a wavelength of 450 nm.

### Simoa assay for tau N-224

The immunoassay (N-224) detecting fragments going from the N-terminal (antibody-binding region: aa 9–18) to aa 224 was developed using single-molecule array (Simoa, Quanterix, Lexington, MA, USA) technology (Fig. 1c). Magnetic beads (Quanterix, Lexington, MA, USA) were conjugated with the capture antibody anti-tau 224 at 0.3 mg/mL according to bead supplier’s conjugation protocol. Prior to each run, Tau 224 recombinant protein calibrator was serially diluted and the biotin-labeled detection antibody Tau 12 (Nordic Biosite) was diluted to 0.5 µg/mL in PBS-Tween with 1% BSA. For each determination, 140,000 assay beads were washed and resuspended in 100 μL of CSF/brain extract, quality control sample, or calibrator. After a washing step, 20 µL of detection antibody was added followed by 30-min incubation. After a final wash, beads were resuspended in 100 µL streptavidin-conjugated β-galactosidase (SBG, Quanterix, Lexington, MA, USA) at 150 pM diluted in SBG Diluent (Quanterix, Lexington, MA, USA). Following 5 min of incubation, the beads were washed and transferred together with resorufin-d-galactopyranoside substrate (RGP, Quanterix, Lexington, MA, USA) to an array of wells, each well only big enough to contain one bead. The array was imaged with a charge-coupled device (CCD) camera imaging system and the images were used to differentiate between empty beads and beads with bound analyte, giving a signal expressed as average enzyme per bead (AEB). To extract concentrations from AEBs, each sample AEB was fitted to a four-parameter logistic curve plotted from the known concentrations of the Tau 224 calibrator run in parallel with the samples. Calibrator points were run in triplicates, while samples were run in duplicates. CSF samples were run neat and brain tissue extracts were run at 100,000-fold dilution.

### ELISA assay for Tau x-224

A second immunoassay (x-224) detecting N-terminally truncated fragments ending at aa 224 (antibody-binding region: aa 159–198) was developed on an ELISA platform (Fig. 1c). 96-well plates were coated and incubated overnight at + 4 °C within house antibody anti-tau 224 at a concentration of 2 µg/mL. After blocking and washing, 75 µL of titrated calibrator (224 recombinant tau fragment) and neat CSF sample were co-incubated with 12.5 µL of mixed biotinylated detection antibody HT7 and BT2 (Thermo Scientific), at a final concentration of 0.0625 µg/mL. After overnight incubation at + 4 °C, the plate was washed and incubated for 30 min with a streptavidin-HRP-conjugated solution (KemEnTek Diagnostics). After a final wash and addition of the chromogen tetramethylbenzidine (TMB ONE, KemEnTek Diagnostics), the plate was read on a Magellan reader at a wavelength of 450 nm.

### Brain tissue samples

All brain lysates were made from cryosectioned fusiform gyrus from 81 AD and 33 controls at Banner Sun Health Institute and classified by Braak stages [16]. This region was chosen due to its close proximity to the entorhinal cortex and higher densities of NFTs than any other neocortical region. Subjects were consented, enrolled and annually assessed in the Arizona Study of Aging and Neurodegenerative Disorders, and then autopsied in the Brain and Body Donation Program [11]. The protocol and consent are annually reviewed by an Institutional Review Board. Brain tissue was collected 5 h or less after death and stored until used at − 80 °C. The cryosections were lysed in RIPA buffer with protease inhibitors (Protease Inhibitor Cocktail tablets, Roche) using the Qiagen TissueLyzer to a final concentration of 2–3 mg/ml, as measured with BCA Protein Assay kit (Pierce). Samples were further diluted to 1:50 for the N-123 and t-tau measurements and to 1:100,000 for the N-224 measurement in their respective assay buffer-containing protease inhibitor (Protease Inhibitor Cocktail tablets, Roche).

### CSF collection and biomarker analyses

All CSF samples were collected by lumbar puncture in the L3/L4 or the L4/L5 inter-space, in the morning. The first 12 mL of CSF were collected in a polypropylene tube and immediately transported to the local laboratory and centrifuged (10 min at 1800*g* at + 20 °C). The supernatant was gently mixed to avoid possible gradient effects, aliquoted in polypropylene tubes, and stored at − 80 °C. CSF t-tau and p-tau concentrations were measured using ELISA (INNOTEST^®^ htau Ag and PHOSPHO_TAU (181P), Fujirebio) as previously described [13, 43]. CSF Aβ1-42 was measured using an ELISA (INNOTEST^®^ Aβ1-42), specifically constructed to measure Aβ starting at amino acid 1 and ending at amino acid 42 [6].

### Discovery CSF cohort

Non-AD subjects (*n* = 20) were patients referred to the hospital for the evaluation of psychiatric or neurological symptoms, but with basic (cell count, albumin ratio, and IgG index) and core (Aβ1-42, t-tau, and p-tau) CSF biomarkers within normal ranges. Patients with AD (*n* = 20) had a clinical diagnosis of AD along with biomarker positive CSF (Supplementary Table 4a). Samples were selected based on biomarker cut-offs for Aβ1-42, t-tau, and p-tau181. Cut-offs used to for a biomarker positive profile was Aβ1-42 < 550 ng/L, t-tau > 400 ng/L, and p-tau > 80 ng/L. The CSF sample aliquots used were de-identified left-over aliquots from clinical routine analyses, following a procedure approved by the Ethics Committee at University of Gothenburg (EPN 140,811).

### Validation CSF cohort

The CSF sample aliquots used were from the Memory Clinic at the University of Lund (Dnr 695/2008). Included participants were characterized as AD (*n* = 46), according to the criteria of National Institute of Neurological and Communicative Disorders and Stroke and the AD and Related Disorders Association [19, 28], and healthy volunteers as the control group (*n* = 50) (Supplementary Table 4b). The applied IWG-2 criterion for allocation to the AD group was a low concentration of Aβ1-42 (< 550 ng/L) with a high level of t-tau (> 400 ng/L) or p-tau181 (> 80 ng/L).

### Longitudinal CSF cohort

Baseline CSF samples from 16 AD, 38 MCI, 20 MCI-AD, and 21 other neurological diseases (OND) subjects were collected at the Center for Memory Disturbances of the University of Perugia (Supplementary Table 5). All patients underwent a baseline clinical examination with a complete neuropsychological assessment and blood chemistry, brain CT, and/or MRI scan to exclude the other causes of cognitive deficit. Subjects were clinically diagnosed according to the NIA-AA AD criteria [28] and Petersen’s MCI criteria [33], with AD CSF biomarkers (Aβ1-42, t-tau, and p-tau) used as support. Cognitive assessment at follow-up was performed through MMSE every 6 months for ~ 2.3 years. OND subjects had no clinical or neuropsychological evidence of cognitive impairment and were diagnosed with Parkinson’s disease (*n* = 15), psychiatric disorder (*n* = 1), white-matter lesions (*n* = 2), Miller–Fisher syndrome (*n* = 1), epilepsy (*n* = 1), and paraneoplastic syndrome (*n* = 1). The study was conducted according to the Helsinki Declaration and approved by the local ethics committee (Prot. N. 19369/08/AV).

### PSP and CBS CSF cohort

Patients with parkinsonism were diagnosed at the Department of Neurology, Movement Disorders Unit, Sahlgrenska University Hospital, Gothenburg, Sweden, by movement disorders specialists. Thirty-one subjects were diagnosed with probable or definite PSP according to the criteria from the National Institute of Neurological Disorders and Stroke and Society for Progressive Supranuclear Palsy (NINDS-SPSP) [27]. Fifteen subjects were diagnosed with probable CBS according to the criteria from Armstrong et al. [7]. All patients underwent lumbar puncture as part of the diagnostic work-up. CSF biomarkers Aβ1-42, t-tau, and p-tau were analyzed at the Clinical Neurochemistry Laboratory, Sahlgrenska University Hospital, Mölndal, Sweden (Supplementary Table 6). Based on the concentrations of Aβ1-42, subjects were further divided in Aβ positive (Aβ+) and Aβ negative (Aβ−). The cut-off applied for an Aβ+ profile was the same as the discovery and validation cohorts (Aβ < 550 ng/L). Aβ- subjects were 15 in the PSP and seven in the CBS group. Concentrations of N-123 and N-224 were compared to the ones in the AD and control groups of the validation cohort; the ratio between QC samples from the different runs was used for normalization. The study was conducted according to the Helsinki Declaration and the collection of samples was approved by the Regional Ethical Board at the University of Gothenburg (*n*. 460–13, 462–95, and 210–95).

### Biomarker stability study CSF cohort

CSF samples were collected during a 6-month multicenter open study on 51 patients with AD on continuous treatment with acetylcholine esterase (AChE) inhibitors, as previously described (Supplementary Table 7) [14, 23]. Briefly, all patients underwent a thorough clinical investigation which included medical history, physical, neurological, and psychiatric examinations. AD was diagnosed following NINCDS-ADRDA criteria [29]. Exclusion criteria were: prominent frontal lobe symptoms, clinical or brain imaging signs of cerebrovascular disease, family history of dementia, treatment with lithium, warfarin, memantine, antidepressants, and neuroleptics. MMSE was performed at baseline to select only mild to moderate severity (MMSE score > 15) AD cases. Treatment with AChE inhibitors at stable doses had to be ongoing for at least 3 months prior to the study. Within 4 weeks after enrolment, subjects underwent cognitive tests MMSE and Alzheimer’s disease Assessment Scale, Cognitive subscale (ADAS-Cog), and CSF tapping. Six months later, cognitive tests were repeated and a second CSF sample was taken. All patients gave informed consent to participate in the study. The study was conducted according to the Helsinki Declaration and approved by the ethics committee at the University of Lund and Uppsala and at the Karolinska Institute, Stockholm, Sweden.

### Isolation of NDEV and PDEV

EVs were isolated from human serum following a protocol previously described, with some alterations [31]. Briefly, 0.5 mL of serum from four AD CSF biomarker positive and four AD CSF biomarker negative subjects were collected and frozen. Cut-offs used to for a biomarker positive CSF profile were Aβ1-42 < 550 ng/L, t-tau > 400 ng/L, and p-tau > 80 ng/L. After thawing, 500 μl of Dulbecco’s solution were added to the samples. Samples were mixed, left for 5 min at RT, and then centrifuged at 4000×*g* for 20 min at 4 °C. Supernatants were transferred to new tubes and mixed with 252 μl Exoquick^®^ exosome precipitating solution (System Biosciences). Samples were incubated for 60 min at 4 °C and then centrifuged at 1500*g* for 20 min at 4 °C. Supernatants were discarded after centrifugation and the pellet was re-suspended in 0.5 ml dH_2_O. Samples were incubated for 1 h at 4 °C with 4 μg in 50 μL of 3% BSA of mouse anti-human CD171 biotinylated antibody (eBio5G3) directed to neuronal L1 cell adhesion molecule (L1CAM). Next, 15 μL of streptavidin-agarose Ultralink resin (Thermo Scientific) was added with 25 μL of 3% BSA and incubated for 30 min at 4 °C with continuous mixing. Samples were centrifuged at 200*g* for 10 min at 4 °C and the supernatants were saved for the analysis of PDEV, while the pellet was re-suspended in 200 μL of 0.1 M glycine HCl by mixing for 10 s and centrifuged at 4500*g* for 10 min at 4 °C to detach L1CAM + EVs from the beads. Supernatants were transferred to new tubes and 25 μL 3% BSA and 15 μL of 1 M Tris–HCl was added. Exosomes were lysed by addition of 260 μL mammalian protein extraction reagent (M-PER; Thermo Scientific) and went through two freeze thaw cycles. Samples were stored at − 80 °C pending immunoassay analysis.

### Statistical analysis

GraphPad Prism v7.02 (GraphPad Software, Inc., La Jolla, CA, USA) was used for the statistical analysis of discovery, validation, longitudinal, and PSP/CBS cohorts. Data were not normally distributed, so the Mann–Whitney and Kruskal–Wallis tests were used for the comparison of the groups. The significant threshold was set to *p* < 0.05. Correlation was tested used the Spearman rank correlation coefficient. Although non-parametric tests were used, linear regression lines were added to the figures as guidance. SPSS v25.0 (IBM Corp., Armonk, N.Y., USA) was used to fit the generalized linear model in the longitudinal cohort. Baseline MMSE values (intercept) and rate of change (slope) were used as outcomes in linear regression models, with CSF N-123 and N-224 tau as predictors. Subjects were divided into quartile groups according to CSF N-123 and N-224 levels at baseline and linear regression models between the quartile groups were compared. In the biomarker stability study, statistical analysis was carried out using the R programming language (version 3.4.3), and all tests were two-sided with a significance threshold set to *p* < 0.05. There were no corrections made for multiple comparisons, so any *p* values close to 0.05 should be considered exploratory. Change in tau N-224 across treatments was tested with non-parametric Kruskal–Wallis test. Post hoc analysis of differences in biomarker concentration between baseline and follow-up and between specific groups was carried out with the Mann–Whitney *U* test. Differences in biomarker concentration across varying number of copies of the ε4 allele of the *APOE* gene were also tested in this way. Differences in biomarker concentration across treatments depending on *APOE* ε4 genotypes were tested by fitting a linear model with an interaction term between treatment and ε4 copies, while both age and sex were included as covariates. The Spearman rank correlation coefficient was used to assess the relationship between change in biomarker concentration and age, sex, and change in treatment outcomes as measured by the MMSE and ADAS-Cog scales.

## Results

### Semi-tryptic tau peptides reveal endogenous cleavage sites

**Fig. 2:**
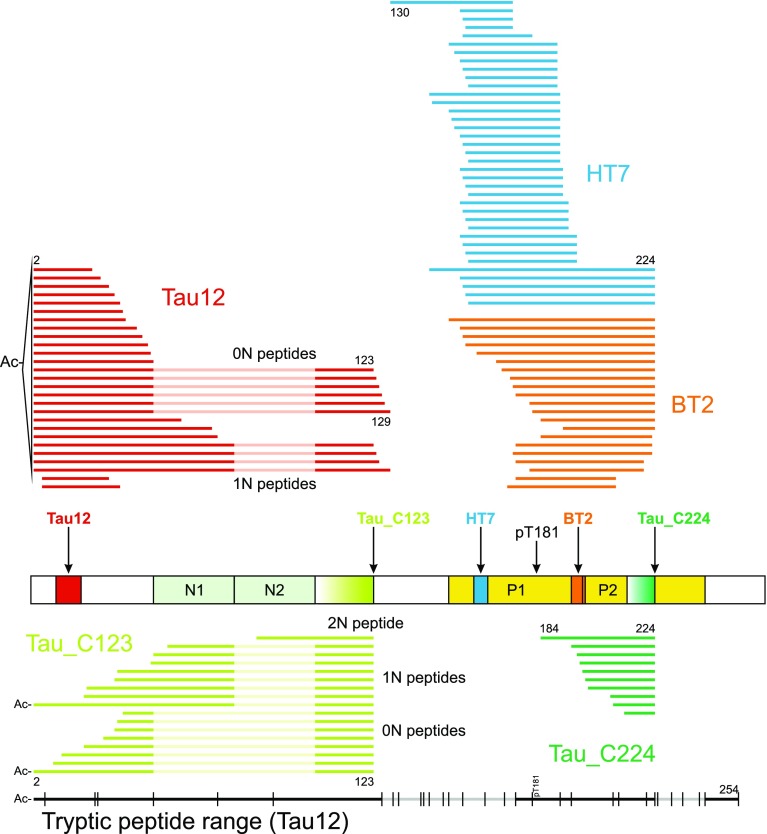
Top: endogenous tau species found with N-terminal and mid-region-specific antibodies (Tau 12 in red, HT7 in blue, BT2 in orange) and neo-epitope-specific antibodies (Tau_C123 in lime, Tau_C224 in green) with selected amino acid positions indicated. Alignment and numbering refer to the 2N isoform; for peptides originating from the 0N and 1N isoforms, 2N sequence portions not included are dimmed. The protein schematic structure shows the respective antibody epitopes and T181. Bottom: in black, range (Ac-2-254) of tryptic peptides detected in CSF with Tau12 (regions with no coverage are dimmed). Vertical lines indicate possible tryptic cleavage sites

**Fig. 3:**
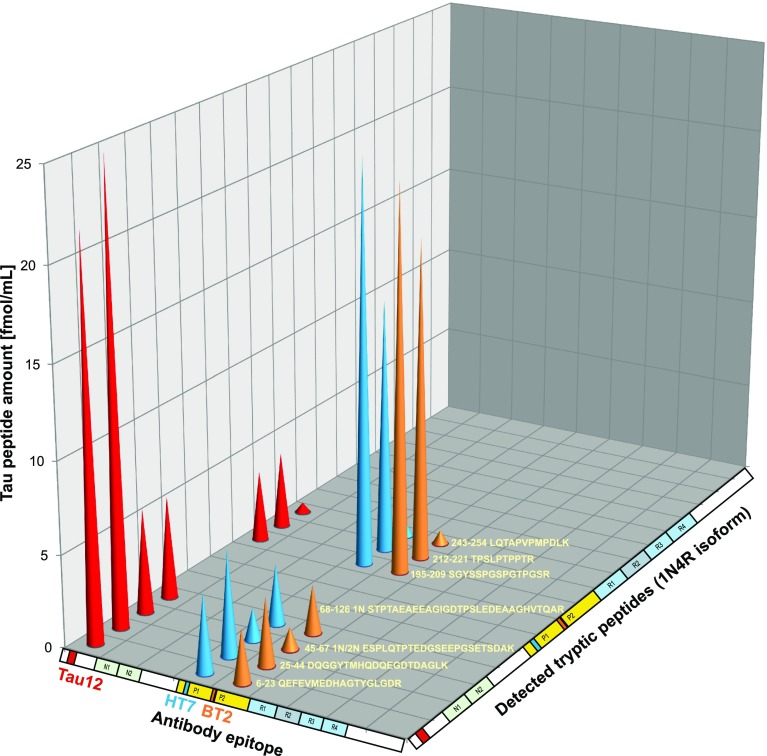
Representation of quantitative MS data of tau fragments immunoprecipitated with Tau 12, HT7, and BT2

Tau was enriched from both brain tissue homogenates and CSF using immunoprecipitation (IP) with Tau12 and treated with trypsin. In brain tissue, tryptic peptides could be detected spanning the whole tau protein, i.e., from the N-terminus (acetylated aa 2) to the C-terminus (aa 441). Peptides representing all isoforms (0N, 1N, 2N, 3R, and 4R) were detected. In CSF, only peptides representing the N-terminal half of tau were detected; the most C-terminal peptide ended at aa 254 and isoform-specific tryptic peptides originating from 0N, 1N, and 2N tau isoforms were detected (Fig. 2, Supplementary Table 1). Peptides phosphorylated at threonine 181 were detected both in brain tissue and in CSF. Furthermore, in both brain and CSF, a number of semi-tryptic peptides, i.e. peptides with either the N- or C-terminus generated by cleavage by trypsin and the other end by endogenous proteases, were identified. Among these were peptides ending at aa 123, 124, and 125, which led us to investigate non-trypsinated samples containing endogenous tau fragments in CSF. We also investigated which tau species were immunoprecipitated when using antibodies with different binding epitopes on tau (Supplementary Table 2). Quantitative data were obtained by spiking a stable-isotope-labeled tau 1N4R protein into the CSF prior to IP. When performing IP with Tau12, tryptic peptides were abundant in the region close to the antibody epitope, while intensities decreased when moving in the C-terminal direction (Fig. 3). For HT7 and BT2, a similar situation was observed; the abundance was high close to the epitopes but decreased markedly at the C-terminal side, but not as clearly toward the N-terminus (Fig. 3). Together, these data indicate that there is an abundance of shorter tau species present in CSF, both at the N-terminus and in the mid-region around aa 150–200. There are also longer species, but it appears that, shortly after aa 221, the abundance drops significantly, and C-terminally of aa 254 no tryptic peptides were detected when using these three antibodies (Fig. 3).

### Endogenous tau fragments ending at aa 123 and 224 are prominent in CSF

Analyses of endogenous tau peptides were performed without tryptic digestion, to preserve the naturally occurring peptides in CSF. Using IP-MS with Tau12, 27 different endogenous tau fragments were identified by MS/MS (excluding oxidized variants at the three available methionine, Met). The majority of these peptides contained the N-terminus of tau protein. The N-terminal peptides were lacking the Met1 amino acid, and were acetylated at the N-terminus. Endogenous species from both the 0N and 1N isoforms were observed, ranging up to aa 129 and including peptides ending C-terminally at aa 123 (Fig. 2 and Supplementary Table 3). With HT7 and BT2, 37 and 21 different mid-region species were identified, respectively, ranging from aa 130 to aa 224. With HT7, which recovers both T181 and pT181 forms, additional peptides phosphorylated at T181 were observed. From the detected peptides from the tau mid-region, it was notable that a major cleavage site was situated between aa 224 and aa 225. The top portion of Fig. 2 shows a summary of the endogenous tau species identified.

### Newly developed neo-epitope antibodies are specific against aa 123 and aa 224

A total of 17 tau species, all ending C-terminally at aa 123 and belonging to all three isoforms (0N, 1N, and 2N), were identified when performing IP-MS of CSF using the anti-tau 123 clone “Tau_1″. Supplementary Fig. 1a shows the MS/MS data for the tau peptide Ac-2–63…103–123 from the 1N isoform (the gap between 63 and 103 is because aa 64–102 are present only in the 2N isoform). Similarly, when performing IP-MS of CSF using the anti-tau 224 clone “Tau_F”, ten different tau species ending C-terminally at aa 224 were detected. Supplementary Fig. 1b shows the MS/MS data for the tau peptide 197–224. The bottom portion of Fig. 2 and Supplementary Table 3 shows a summary of the endogenous tau species detected when using these two antibodies.

### Anti-224 antibody (but not anti-123) stains neurofibrillary tangles

**Fig. 4:**
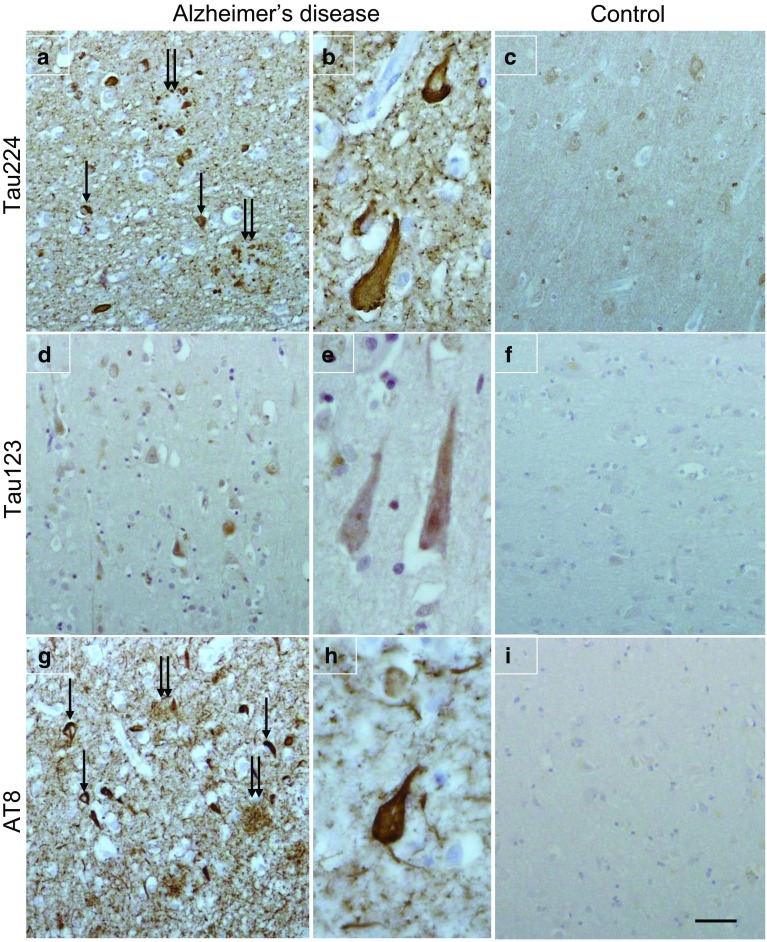
Tau immunohistochemistry in Alzheimer’s disease (**a** and **b**; **d** and **e**; **g** and **h**) and a neurologically normal control (**c**, **f**, and **i**). Anti-tau 224 immunohistochemistry shows neurofibrillary tangles (**a**, arrow) and dystrophic neurites (**a**, double arrow) surrounded by neuropil threads. Neurofibrillary tangles show at higher magnification (**b**). Faint punctuate neuronal cytoplasmic staining was observed in the normal control. Neurons in AD are weakly positive for anti-tau 123 and negative in the normal control. For comparison, sequential sections were immunohistochemically stained with AT8 to show the presence of neurofibrillary tangles in AD (**g**, arrow), dystrophic neurites (**g**, double arrow), and filamentous structure of the neurofibrillary tangles (**h**). AT8 staining is absent in the normal control. Bar (in **i**) represents 50 µm in **a**, **c**, **d**, **f**, **g**, and **i**; 20 µm in **b**, **e**, and **h**

Immunohistochemical analysis with anti-tau 224 antibody demonstrated neurofibrillary tangles and dystrophic neurites surrounded by neuropil threads, whilst faint punctate neuronal cytoplasmic staining was observed in the normal control (Fig. 4a–c). The anti-tau 123 antibody showed weak neuronal cytoplasmic staining in AD which was negative in the normal control (Fig. 4d–f). Immunohistochemical staining on sequential sections with anti-AT8 shows the presence of neurofibrillary tangles in AD and dystrophic neurites (Fig. 4g) and the filamentous structure of the neurofibrillary tangles (Fig. 4h). No AT8 staining was present in the normal control (Fig. 4i).

### N-224 tau and t-tau concentrations show a decreasing trend in AD brain samples along Braak stages

**Fig. 5:**
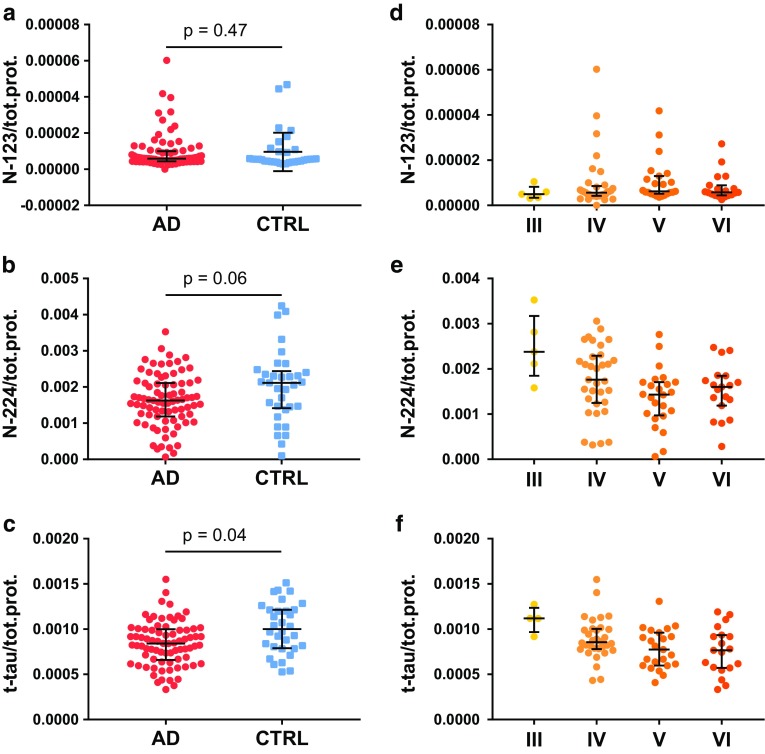
Brain levels (normalized for total protein content) of N-123 (**a**), N-224 (**b**) fragments, and t-tau (**c**) in Alzheimer’s disease and controls and in AD over Braak stages III–VI (**d**, **e**, **f**) (lines representing the median, bars representing interquartile range)

There was no significant difference in the concentration between AD and controls for the N-123 fragment (*p* = 0.5) (Fig. 5a). The N-224 tau fragment was present in AD brain at significantly lower concentrations compared to controls (*p* = 0.01), although not significant when normalized to total protein content (*p* = 0.06) (Fig. 5b). T-tau measurement shows significantly lower concentrations in AD compared to controls both for absolute and normalized concentrations (*p* = 0.01 and *p* = 0.004, respectively) (Fig. 5c). When dividing the samples by Braak staging, the N-224 fragment and t-tau tended to decrease from stage I to VI when including all samples and in AD samples from stage III–VI (Fig. 5e). No visible trend is present for the fragment N-123 (Fig. 5d) or for any of the tau fragments in controls.

### N-123 and N-224 tau concentrations in CSF are significantly increased in AD in a discovery cohort

The CSF concentration of tau N-123 was significantly higher in AD compared to non-AD subjects (*p* < 0.001) (Supplementary Fig. 2a). There was a significant positive correlation between the N-123 fragment and t-tau in the AD group (*r* = 0.56, *p* = 0.01) but not in the control group (*r* = 0.15, *p* = 0.58). Similarly, the concentration of the tau N-224 was significantly higher in AD compared to controls (*p* < 0.001) (Supplementary Fig. 2b). Furthermore, a significant positive correlation was found between tau N-224 tau and t-tau in the control group (*r* = 0.65, *p* = 0.004, *α* < 0.05) but not in the AD group (*r* = 0.432, *p* = 0.1308). N-123 and N-224 showed a positive correlation in AD (*r* = 0.9, *p* < 0.0001) but not in controls. A significant correlation was also found between N-224 tau and N-mid domain tau (N-mid, aa 9–198) in AD (*r* = 0.72, *p* = 0.01) as well as in controls (*r* = 0.69, *p* = 0.01).

### N-224 and x-224 tau (but not N-123) concentrations in CSF are significantly increased in AD in a validation cohort

**Fig. 6:**
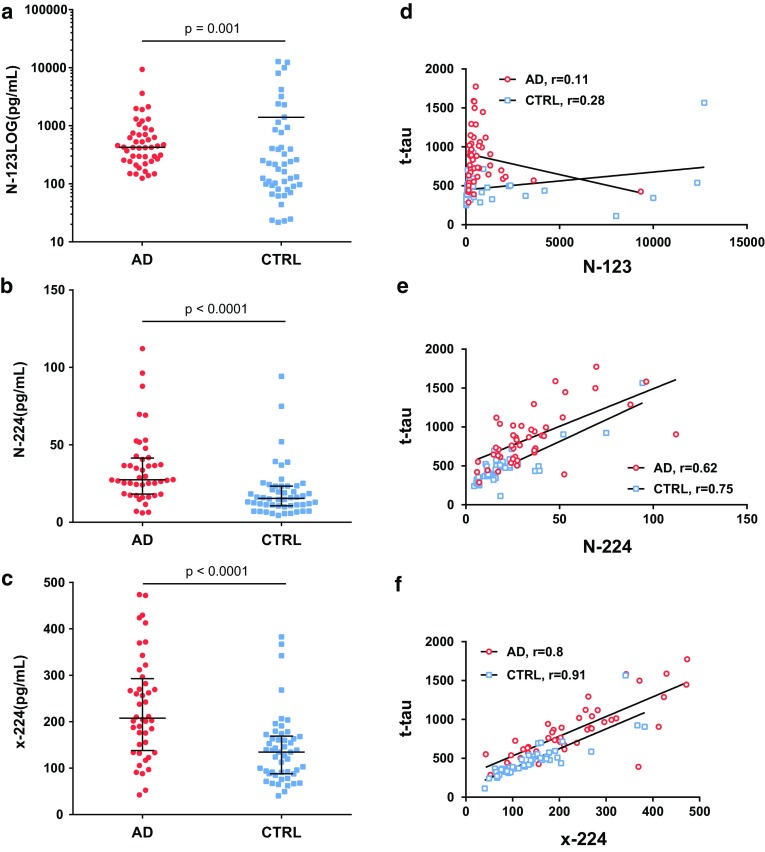
Validation cohort. Left column: concentration range of N-123 (**a**), N-224 (**b**), and x-224 (**c**) fragments in AD and controls (lines representing median; bars representing interquartile range; in a, bars removed due to the LOG scale). Right column: correlation of N-123 (**d**), N-224 (**e**), and x-224 (**f**) fragment to t-tau and linear regression in AD and controls

For tau N-123, we observed an opposite behaviour compared to the discovery cohort, with significantly higher concentrations in controls vs. AD (*p* = 0.001) (Fig. 6a). N-123 concentrations did not correlate with t-tau (Fig. 6d). In contrast, tau N-224 was significantly higher in patients with AD compared to controls (*p* < 0.001) (Fig. 6b). Tau N-224 correlated to t-tau in both AD and controls (*r* = 0.62 vs. *r* = 0.75) and the correlation was significant in both groups (*p* < 0.0001) (Fig. 6e). N-123 and N-224 had a positive correlation in controls (*r* = 0.5, *p* < 0.0001) but not in AD. N-224 also shows a significant correlation to N-mid domain tau (N-mid, aa 9–198), (*r* = 0.81 and *r* = 0.76 for AD and controls, respectively, *p* < 0.0001 for both). Tau x-224 concentration was significantly higher in AD CSF samples compared to controls (*p* < 0.0001) (Fig. 6c). Tau x-224 correlates positively to t-tau in AD and control cohorts (*r* = 0.8 and 0.9, respectively) and both correlations are significant (*p* < 0.0001) (Fig. 6f).

### N-123 and N-224 tau baseline concentrations in CSF are related to severity of cognitive impairment in a longitudinal cohort

**Fig. 7:**
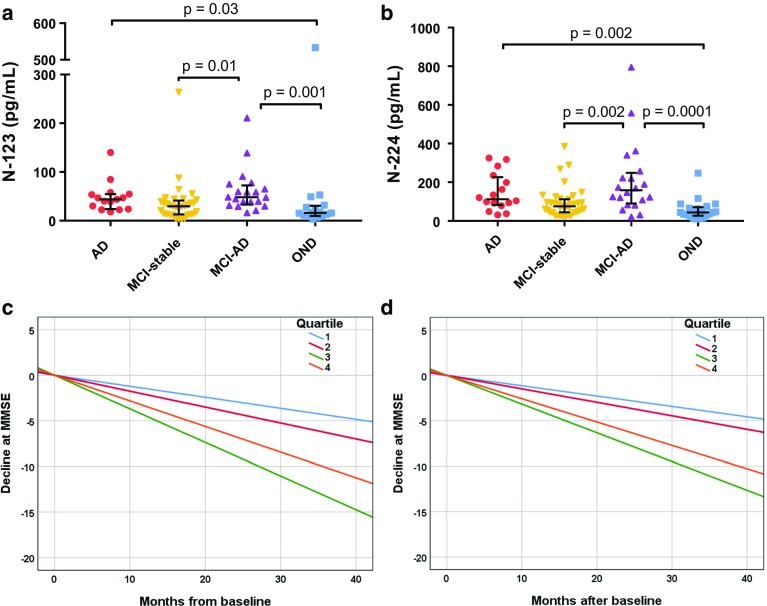
Longitudinal cohort. **a**, **b** Concentration range of N-123 (**a**) and N-224 (**b**) fragments in AD, MCI, and MCI-AD and OND (lines representing the median, bars representing interquartile range). **c**, **d** Disease progression over time in AD, MCI-AD and MCI cohorts, as measured by change in MMSE scores. Higher levels of CSF N-123 (**a**) and N-224 (**b**) tau at baseline are related to faster decline. N-123: Quartile (Q) 1: *n* = 16, Q2: *n* = 15, Q3: *n* = 15 and Q4: *n* = 16; N-224: Q1: *n* = 17, Q2: *n* = 17, Q3: *n* = 17, and Q4: *n* = 17

Baseline levels of the N-123 and N-224 fragments were significantly higher in AD vs. OND (*p* = 0.03 and 0.002, respectively), mild cognitive impairment due to AD (MCI-AD) vs. MCI (*p* = 0.01; *p* = 0.002), and MCI-AD vs. OND (*p* = 0.001; *p* < 0.0001) (Fig. 7a, b). Group differences for N-123 and N-224 did not change significantly when controlling for age and gender effects (*p* > 0.05). N-123 and N-224 showed a positive correlation in MCI (*r* = 0.59, *p* = 0.001), MCI-AD (*r* = 0.61, *p* = 0.004), and OND group (*r* = 0.67, *p* = 0.005), but not in AD. Based on the baseline CSF N-123 and N-224 tau levels, AD, MCI-AD and MCI groups were classified into quartiles (Q1, Q2, Q3, and Q4), which were tested for association with disease progression as examined by longitudinal change in Mini-Mental State Examination (MMSE). MMSE score decreased significantly over years from baseline for the highest quartile (Q4) for both fragments (N-123: adj. *R*^2^ = 0.36, *β* = − 0.61, *p* = 0.0001; N-224: adj. *R*^2^ = 0.34, *β* = − 0.58, *p* = 0.0002) (Fig. 7c, d). A significant decrease at MMSE score in Q4 was not observed for t-tau and p-tau (*p* > 0.05).

### N-224 tau does not correlate to t-tau and p-tau in PSP and CBS

**Fig. 8:**
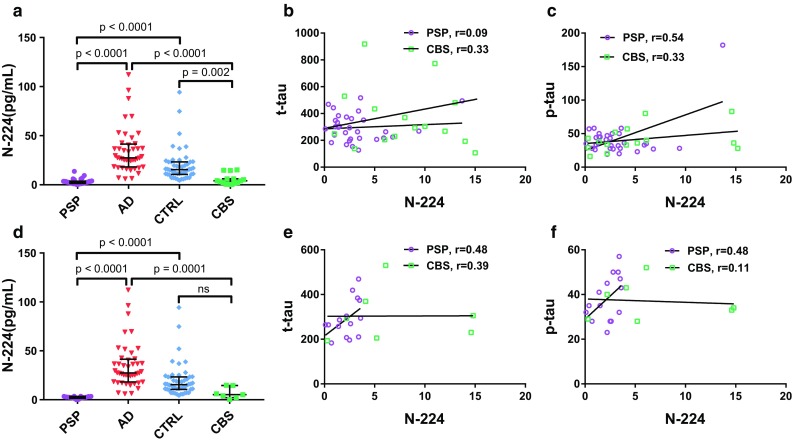
PSP and CBS cohort. **a**, **d** Concentration of N-224 tau in PSP and CBS (**a**: Aβ+ and Aβ; **d**: Aβ− only) compared to AD and controls from the validation cohort (lines representing the median, bars representing interquartile range). **b**, **c**, **e**, **f** Correlation to t-tau (**b**) and p-tau (**c**) and linear regression in PSP and CBS cohorts (**b**, **c**: Aβ+ and Aβ; e, f: Aβ− only)

CSF levels of N-224 tau were significantly lower in PSP and CBS vs. both AD (*p* < 0.0001, both) and controls (PSP: *p* < 0.0001; CBS: *p* < 0.002) (Fig. 8a). CSF N-224 tau concentrations did not significantly correlate to t-tau in both PSP (*r* = 0.09) and CBS (*r* = 0.33) (Fig. 8b). Correlation to p-tau was significant in PSP (*r* = 0.54, *p* = 0.002) but not in CBS (*r* = 0.33) (Fig. 8c). After removing the Aβ+ subjects, no significant correlation with t-tau or p-tau was present in any of the groups (PSP: *r* = 0.48 for both: CBS: *r* = 0.39, *r* = 0.11) (Fig. 8e–f). Concentrations of N-224 tau in Aβ− PSP were significantly lower than in AD and controls (*p* < 0.0001), while, in CBS, concentrations were significantly lower only when compared to AD (*p* = 0.0001) (Fig. 8d). N-123 tau concentrations were below LLOQ in 8 out of 15 (53.3%) CBS cases and 19 out of 31 (61.3%) PSP cases.

### N-224 tau concentrations in CSF are not affected by treatment with AChE inhibitors

Levels of tau N-224 did not significantly differ between baseline and follow-up (*p* = 0.78), with an average CV of 13.4% and 12% change (Supplementary Fig. 3a, b, c). The concentrations were not significantly different across drug treatments (*p* = 0.08). There were no significant differences between specific groups when comparing donepezil to rivastigmine treatment (*p* = 0.28) and donepezil to galantamine (*p* = 0.37). Moreover, N-224 tau levels did not differ significantly depending on number of *APOE* ε4 copies (*p* = 0.48). There was no interaction effect between treatment and *APOE* ε4 copies for tau N-224 (*p* = 0.35). There was no strong association between change in tau N-224 and either change in MMSE (*r* = − 0.12, *p* = 0.38) or change in ADAS-Cog (*r* = 0.03, *p* = 0.81). Tau N-224 did not differ across sex (*p* = 0.22), or age (*p* = 0.08).

### N-224 tau specifically present in NDEV and N-123 tau in both NDEV and PDEV

**Fig. 9:**
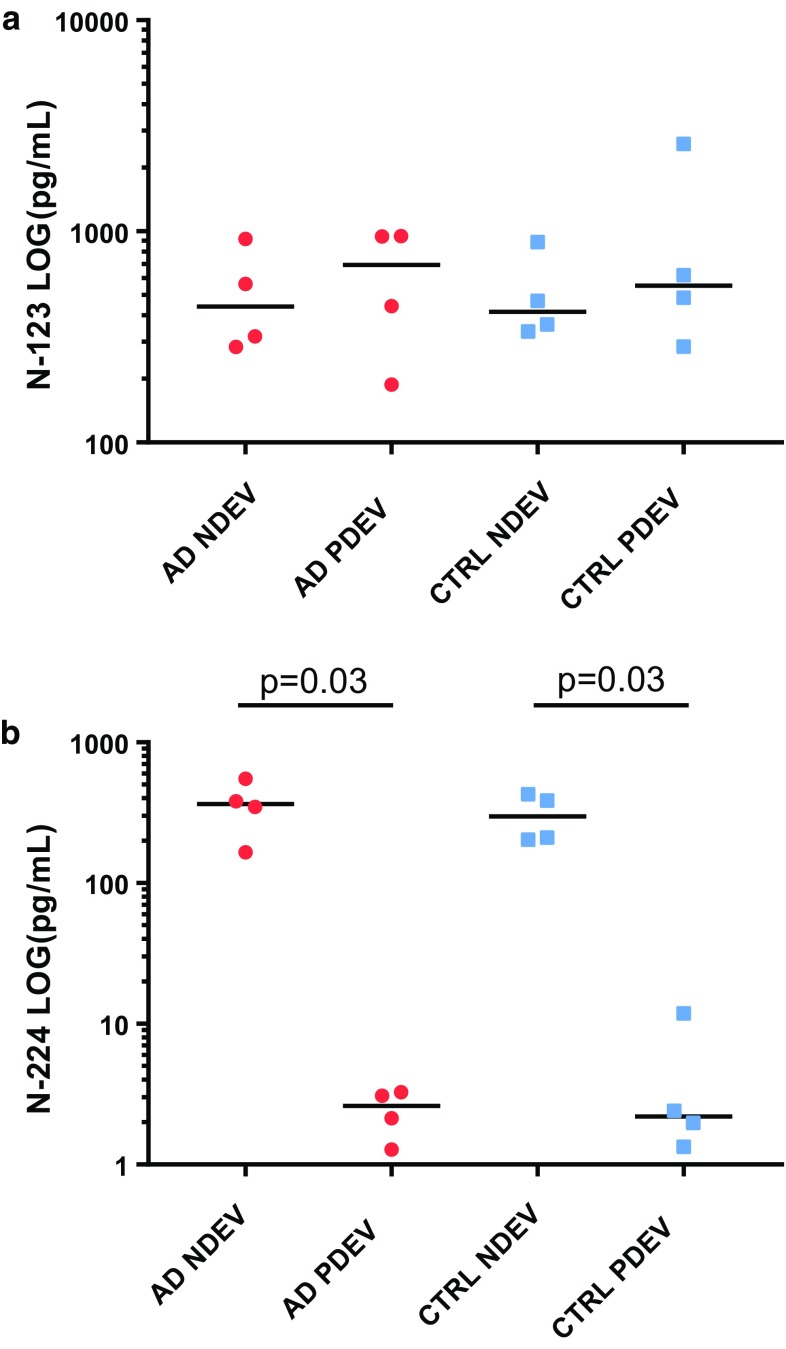
Scatter dot plots of N-123 (**a**) and N-224 tau (**b**) concentrations in serum extracellular vesicles (EVs) lysates, divided in AD and control neuronally derived EVs (NDEV) and peripherally derived EVs (PDEV) samples (lines representing median)

The N-123 tau fragment was measurable in all EV lysates with no significant difference between NDEVs, i.e., L1CAM-positive samples, and peripherally derived EVs (PDEVs), i.e., supernatants left after L1CAM-positive EVs had been bound to the beads. There were no differences comparing levels of tau N-123 in NDEVs or PDEVs comparing AD and control samples. Comparable concentrations of the fragments were observed within and between cohorts (Fig. 9a). Conversely, the N-224 tau fragment was significantly higher in NDEV lysates compared to PDEV lysates (*p* = 0.03) in both AD and control groups. Fold differences between average concentrations of N-224 tau in NDEV samples compared to PDEV were 148 and 70 in the AD and control group, respectively. Differences between AD and control NDEV samples were not significant (Fig. 9b).

## Discussion

Using IP-MS analysis with anti-tau antibodies (Tau 12, HT7, and BT2), we collected quantitative data demonstrating several tau fragments in CSF, both at the N-terminus and in the mid-region (Fig. 2). Shortly after aa 221, the abundance of the fragment intensities dropped significantly and C-terminally of aa 254 no tryptic peptides were detected when using these three antibodies (Fig. 3). The two major tau pools detected were composed of peptides ending at aa 123 and aa 224, respectively, suggesting prominent proteolytic cleavage of the protein around these sites. It should be noted that the absence of even longer peptides corresponding to the data from trypsinated CSF samples is likely a methodological bias; MS favors detection and identification of smaller peptides and there are likely longer tau variants present in CSF which evade identification with the LC–MS configuration used in the current study. Furthermore, the choice of a C18 column for separation may also have contributed to the bias against longer peptides.

In support of the findings in the present study, the previous studies in brain tissue identified N-terminally truncated fragments starting from aa 124 and proteomics studies on CSF confirmed the presence of fragments ending at aa 224 [9, 18, 35]. We, therefore, generated antibodies against these specific fragment epitopes and confirmed their specificity through ELISA cross-reactivity tests as well as IP-MS. The antibodies were successfully tested for immunohistochemistry on AD and control brain slices, and used to develop targeted immunoassays. Validation of immunoassays on different platforms (ELISA, Simoa), and their application on exosome lysates, soluble fraction of brain extracts and CSF from four independent cohorts of AD and control subjects, showed that the novel assays were specific, sensitive, and robust, with fragment concentrations within the calibration range.

Immunohistochemical staining showed positivity to the anti-tau 224 antibody in neurofibrillary tangles and neuropil threads (Fig. 4a, b), suggesting that this fragment is deposited in AD and colocalized with the tangles. Data from brain extracts showed an apparent decreasing trend for the N-224 tau fragment over the course of disease (Braak III–VI), with lower concentration in AD brain compared to control, although the difference was not statistically significant when normalized to total protein content (Fig. 5b, e). T-tau shows the same behaviour in brain extracts, being significantly lower in AD brain compared to controls, even when normalized to total protein content (Fig. 5c, f). Although it might appear counterintuitive, this behaviour is justified by the technique used for extraction: RIPA buffer extracts all of the fractions soluble in weak detergents and also a proportion of tau insoluble in no-detergent or non-ionic detergent conditions, but not tau aggregated into tangles, as it would require additional extraction steps. These findings suggest that N-224 might be trapped in the fuzzy coat of tau tangles and, therefore, less likely to be extracted with this technique in samples from later stages of disease, although these observation need to be confirmed with further experiments, i.e., sarkosyl extraction and treatment of the sarkosyl-insoluble pellet. On the other hand, in CSF the N-224 tau fragment shows an opposite behaviour to the brain, being significantly higher in AD CSF compared to control and in MCI-AD compared to stable MCI (Figs. 6b, 7b). Thus, brain and CSF give us complementary information about tau metabolism in AD compared to controls.

It seems, therefore, that, in the CNS, there is a disease-related upregulated cleavage at this site which leads to enhanced release from neurons of the N-terminal fragment, ultimately leading to higher CSF concentration of the fragment and a deposition into tangles. At a tangle level, the 224-cleaved tau might, therefore, be an indirect marker of ongoing tangle formation. Another potential explanation for the results (with similar practical implications regarding the possible association of the fragment with tangle pathology) is that the production of the fragment could be enhanced in the fuzzy coat of tangles. These promising results open up for the investigation of tau fragments in tangle pathology, using various techniques, such as laser capture of tangles and more refined extraction techniques.

Several studies have been published on N-terminal cleavage of tau from proteases of the calpain and caspase family [20, 25]. The N-224 might also represent the neurotoxic N-terminal tau fragment of 20-22 kDa that has been shown by western blot in several studies [1–4, 8]. Other studies have also shown the presence of N-terminal fragments ranging from 20 to 40 kDa in CSF [30]. Secretion of N-terminal tau fragments has also been previously described [36]. Further studies to establish which enzyme is responsible for this cleavage and whether this activity is specifically upregulated in AD are needed.

The fragment x-224 proved to be less interesting than N-224, meaning that it more tightly correlated with t-tau in AD and controls, probably due to the overlapping region of interest of the assays used to measure the two tau entities (aa 159–240). Tau N-224, on the other hand, might represent the product of a different metabolic route affecting the N-terminus of the protein as opposed to the mid-region pool.

When analyzing baseline CSF values of N-224 tau in subjects monitored over time by means of MMSE, we observed that the magnitude of cognitive decline is related to the initial concentration of the fragment (Fig. 7d). It seems, therefore, that not only the N-224 fragment is AD-related, but its concentration could also have a predictive value of worse cognitive outcome. In the previous studies, no correlation was found between baseline core CSF biomarker levels and follow-up cognitive scores [39]. One limitation of our study is that the subjects were monitored by MMSE, which is not the optimal test to detect the early AD-related changes in cognition [40]. Future studies on prognostic value of tau fragments should be performed in longitudinal cohorts where cognitive assessment at follow-up includes ADAS-Cog and delayed word recall [34].

When comparing AD to primary tauopathies PSP and CBS, we observe that CSF N-224 tau concentrations are significantly decreased in PSP and CBS, being even lower than in controls. We have also measured core AD CSF biomarkers to discriminate AD-like PSP and CBS. Even after excluding CSF Aβ+ subjects, the same behaviour is observed, with the loss of significant difference between CBS and controls possibly due to a statistical power issue (seven CBS cases vs. 50 controls). A similar behaviour in PSP and CBS has been observed before for t-tau and p-tau [21, 41, 42]. This might be due to an overall lower concentration of CSF tau in the primary tauopathies, and it is not known whether this is due to reduced tau production, reduced secretion into the CSF, or both. Difference in the distribution of tau pathology between neurons (AD) and glia (PSP and CBD) might also account for this behaviour. The lack of correlation of N-224 tau to p-tau and t-tau suggests a different underlying disease mechanism compared to AD, which does not include cleavage of N-terminal tau. Parallel investigation of N-224 tau, t-tau, and p-tau in PSP and CBS could help to understand these different mechanisms and add to the information from classic CSF tau biomarkers.

In terms of stability and predictive power, it is reassuring that the levels of N-224 tau in AD subjects were basically unaffected by AChE inhibitor treatment at 6-month follow-up, regardless of *APOE* status, age, gender, or drug type. This type of study would benefit from a longer follow-up, to evaluate possible long-term oscillations in the concentration and association with cognitive scores.

Immunohistochemical data on N-123 tau showed only a faint positivity of neuronal staining with anti-tau 123 and mostly in the cytoplasm, which makes the presence of N-123 tau in the tangles unlikely and reinforces the idea of an active secretion of the fragment (Fig. 4d–f). A similar behaviour for the N-123 fragment was observed in CSF and brain extracts, with a wide range of concentrations in both AD and control cohorts (Figs. 5a, 6a). More precisely, the difference in concentration between the two groups is not consistent throughout the cohorts, and no trend is visible over the course of disease as measured by Braak staging. Although the N-123 fragment does not seem to have a clear diagnostic role, the previous publications suggest that the function of tau is affected by N-terminal shedding and that N-terminal integrity is necessary for microtubule stability, tau solubility, and cell survival [5, 18, 24]. One possible speculation is that this tau fragment might represent a general marker for tau metabolism occurring in CNS and peripheral nervous system, a hypothesis reinforced by its presence in the PDEV. However, no indications have been found on how this relates to the disease state and/or stage, although higher CSF levels at baseline are related to higher rate of cognitive decline at follow-up as measured by decline at MMSE (Fig. 7c). Data from serum EVs do not corroborate the brain or disease specificity of the fragment, showing similar level of N-123 in NDEV and PDEV from AD and controls (Fig. 9a). These findings are opposed to the ones on N-224 tau in serum EV lysate; here, N-224 appears to be brain-specific, showing concentrations close to zero in PDEV, although not being decisive in discriminating between AD and controls (Fig. 9b). It is possible that there is an intrinsic balance between tau fragmentation and stability, and that the N-123 fragment could be a marker of physiological processes within a given range of concentrations. It is also evident that N-123 is more abundant tau fragment than N-224, being readily measureable by ELISA, which is much less sensitive than Simoa. Moreover, no correlation between the concentrations of N-123 and N-224 tau is present in the AD groups of the validation and longitudinal cohorts, while it is present in control, OND, MCI, and MCI-AD groups. The biological mechanism underlying the difference in abundance between N-123 and N-224 is currently unknown, but the aggregated results suggest that the two fragments might reflect different mechanism of tau processing in AD.

In conclusion, our data suggest that, among the several fragments of tau present in CSF, there are two major pools consisting of tau species cleaved at either aa 123 or 224. While cleavage generating tau N-123 could be part of the normal function of tau turnover, the generation of tau N-224 is related to AD. In support of this hypothesis is the lack of consistent differences of CSF N-123 tau levels comparing AD and control, while fragments ending at aa 224 are significantly higher in AD CSF compared to control and relate to the decrease in cognitive performance over time. Future studies evaluating whether these tau cleavages may promote tau aggregation and propagation are of high interest. Furthermore, studies in primary tauopathies show great promise for elucidating the role of differential tau processing in AD compared to the other tauopathies.

## Electronic supplementary material

Below is the link to the electronic supplementary material.

Supplementary material 1 (DOCX 195 kb)
