# Supplementary material for: Novel tau fragments in cerebrospinal fluid: relation to tangle pathology and cognitive decline in Alzheimer’s disease
Source: Acta Neuropathol. 2018 Dec 13;137(2):279–96. doi: 10.1007/s00401-018-1948-2 (PMC6514201; doi:10.1007/s00401-018-1948-2)
Supplement: Supplementary file 1 — Supplementary material 1 (DOCX 195 kb) [file 401_2018_1948_MOESM1_ESM.docx]

Supplementary fig. 1 MS/MS data for the tau 1N peptide Ac-2-63...103-123 (a) and for the tau peptide 197-224 (b) from CSF.

**A**

**B**

Supplementary fig. 2 Discovery cohort. Concentration range of N-123 (a) and N-224 (b) fragments in AD and controls (lines representing the median, bars representing interquartile range).

A B

Supplementary fig. 3 Biomarker stability study cohort. Concentrations at baseline and follow-up (a), % change (b) and %CV (c) of N-224 fragment


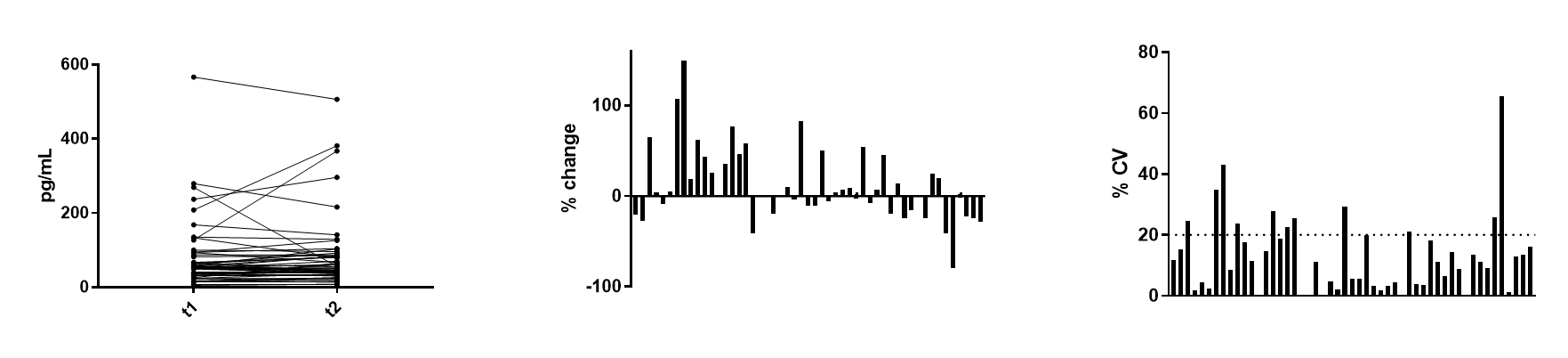


A B C

Supplementary fig. 4 ROC curve for N-224 assay from validation cohort

Supplementary tab. 1 Quantified tryptic tau peptides in CSF.

|  |  | **Estimated concentration [fmol/µL]** | | | | |  |  | |  | |
| --- | --- | --- | --- | --- | --- | --- | --- | --- | --- | --- | --- |
| **Sequence** | **Tau441 numbering** | **Tau12** | **HT7** | **BT2** | **13A6** | **KJ9A** | | |  | |
| QEFEVMEDHAGTYGLGDR | 6-23 | 21.77 | 4.34 | 2.92 | 1.12 | 0.01 | | |  | |
| DQGGYTMHQDQEGDTDAGLK | 25-44 | 24.96 | 5.75 | 3.72 | 1.13 | 0.00 | | |  | |
| ESPLQTPTEDGSEEPGSETSDAK | 45-67 1N/2N | 5.72 | 1.78 | 1.22 | 0.40 | 0.00 | | |  | |
| STPTAE...AEEAGIGDTPSLEDEAAGHVTQAR | 68-73…103-126 1N | 5.64 | 3.31 | 2.67 | 1.65 | 0.02 | | |  | |
| SGYSSPGSPGTPGSR | 195-209 | 3.93 | 22.59 | 21.57 | 9.44 | 0.26 | | |  | |
| TPSLPTPPTR | 212-221 | 4.23 | 14.33 | 17.91 | 13.40 | 0.04 | | |  | |
| LQTAPVPMPDLK | 243-254 | 0.57 | 0.86 | 0.77 | 1.01 | 0.07 | | |  | |
| IGSLDNITHVPGGGNK | 354-369 | 0.03 | 0.03 | 0.03 | 0.04 | 0.59 | | |  | |
| SPVVSGDTSPR | 395-406 | 0.08 | 0.01 | 0.02 | 0.01 | 0.18 | | |  | |
| HLSNVSSTGSIDMVDSPQLATLADEVSASLAK | 407-438 | 0.28 | 0.03 | 0.09 | 0.14 | 0.04 | | |  | |

Supplementary tab.2 Antibody epitopes.

|  | **Epitope (aa)** |
| --- | --- |
| **Tau 12** | 9-18 |
| **HT7** | 159-163 |
| **BT2** | 194-198 |
| **Tau 123** | 123 |
| **Tau 224** | 224 |

Supplementary tab. 3 Identified endogenous tau peptides in CSF.

| **Tau12** | **60 peptides** |  |  |  |  |  |  |
| --- | --- | --- | --- | --- | --- | --- | --- |
| **Peptide** | **Theoretical Mass [Da]** | **Charge** | **Δm [ppm]** | **expect** | **b-ions** | **y-ions** | **Sequence** |
| 2-22 | 2392.0277 | 3+ | -4.2 | 1.70E-06 | 9 | 3 | AEPRQEFEVMEDHAGTYGLGD + acA2 |
| 2-25 | 2791.2507 | 3+ | -3.2 | 2.00E-07 | 10 | 11 | AEPRQEFEVMEDHAGTYGLGDRKD + acA2 |
| 2-25 | 2807.2457 | 4+ | 1.1 | 9.00E-09 | 10 | 15 | AEPRQEFEVMEDHAGTYGLGDRKD + acA2 + oxM11 |
| 2-28 | 3033.3523 | 4+ | -0.2 | 9.80E-11 | 11 | 11 | AEPRQEFEVMEDHAGTYGLGDRKDQGG + acA2 |
| 2-28 | 3049.3472 | 4+ | 0.4 | 4.50E-04 | 8 | 8 | AEPRQEFEVMEDHAGTYGLGDRKDQGG + acA2 + oxM11 |
| 2-30 | 3297.4633 | 4+ | -3.8 | 2.00E-02 | 6 | 1 | AEPRQEFEVMEDHAGTYGLGDRKDQGGYT + acA2 |
| 2-30 | 3313.4582 | 4+ | -2.7 | 2.00E-01 | 5 | 0 | AEPRQEFEVMEDHAGTYGLGDRKDQGGYT + acA2 + oxM11 |
| 2-32 | 3565.5627 | 5+ | 0.1 | 7.60E-13 | 6 | 16 | AEPRQEFEVMEDHAGTYGLGDRKDQGGYTMH + acA2 |
| 2-32 | 3581.5576 | 4+ | -0.2 | 4.40E-10 | 14 | 6 | AEPRQEFEVMEDHAGTYGLGDRKDQGGYTMH + acA2 + oxM11/M31 |
| 2-32 | 3597.5525 | 5+ | -0.3 | 1.20E-06 | 12 | 14 | AEPRQEFEVMEDHAGTYGLGDRKDQGGYTMH + acA2 + oxM11 + oxM31 |
| 2-33 | 3693.6212 | 5+ | -1.0 | 1.10E-08 | 12 | 19 | AEPRQEFEVMEDHAGTYGLGDRKDQGGYTMHQ + acA2 |
| 2-33 | 3709.6162 | 4+ | -3.5 | 1.70E-08 | 12 | 2 | AEPRQEFEVMEDHAGTYGLGDRKDQGGYTMHQ + acA2 + oxM31 |
| 2-33 | 3725.6111 | 4+ | -0.3 | 2.30E-03 | 9 | 3 | AEPRQEFEVMEDHAGTYGLGDRKDQGGYTMHQ + acA2 + oxM11 + oxM31 |
| 2-34 | 3824.6431 | 4+ | -4.4 | 2.90E-03 | 7 | 1 | AEPRQEFEVMEDHAGTYGLGDRKDQGGYTMHQD + acA2 + oxM31 |
| 2-38 | 4253.7927 | 4+ | -3.9 | 1.20E+00 | 3 | 0 | AEPRQEFEVMEDHAGTYGLGDRKDQGGYTMHQDQEGD + acA2 + ox(M31/M11) |
| 2-40 | 4453.8724 | 5+ | 0.5 | 2.50E-09 | 20 | 1 | AEPRQEFEVMEDHAGTYGLGDRKDQGGYTMHQDQEGDTD + acA2 |
| 2-40 | 4469.8673 | 5+ | 1.2 | 1.10E-13 | 23 | 2 | AEPRQEFEVMEDHAGTYGLGDRKDQGGYTMHQDQEGDTD + acA2 + oxM31/(M11) |
| 2-40 | 4469.8673 | 5+ | 0.4 | 3.60E-02 | 7 | 1 | AEPRQEFEVMEDHAGTYGLGDRKDQGGYTMHQDQEGDTD + acA2 + oxM11 + oxM31 |
| 2-42 | 4597.9259 | 4+ | -5.4 | 9.00E-01 | 6 | 0 | AEPRQEFEVMEDHAGTYGLGDRKDQGGYTMHQDQEGDTDAG + acA2 + ox(M31/M11) |
| 2-43 | 4695.0150 | 5+ | 1.4 | 1.90E-07 | 15 | 1 | AEPRQEFEVMEDHAGTYGLGDRKDQGGYTMHQDQEGDTDAGL + acA2 |
| 2-43 | 4711.0100 | 5+ | 4.3 | 4.30E-07 | 14 | 1 | AEPRQEFEVMEDHAGTYGLGDRKDQGGYTMHQDQEGDTDAGL + acA2 + oxM31/(M11) |
| 2-44 | 4711.0100 | 4+ | -4.9 | 7.50E-02 | 9 | 0 | AEPRQEFEVMEDHAGTYGLGDRKDQGGYTMHQDQEGDTDAGLK + acA2 + oxM31/(M11) |
| 5-28 | 2694.2092 | 3+ | -0.3 | 5.50E-04 | 6 | 5 | RQEFEVMEDHAGTYGLGDRKDQGG |
| 5-32 | 3242.4146 | 4+ | -0.6 | 1.00E-05 | 11 | 5 | RQEFEVMEDHAGTYGLGDRKDQGGYTMH + oxM11/M31 |
| 5-32 | 3258.4095 | 4+ | 0.0 | 3.90E-02 | 5 | 1 | RQEFEVMEDHAGTYGLGDRKDQGGYTMH + oxM11 + oxM31 |
| **0N** |  |  |  |  |  |  |  |
| 2-44...103-123 | 6873.0227 | 6+ | 1.0 | 5.70E-25 | 43 | 23 | AEPRQEFEVMEDHAGTYGLGDRKDQGGYTMHQDQEGDTDAGLK...AEEAGIGDTPSLEDEAAGHVT + acA2 |
| 2-44...103-123 | 6889.0176 | 5+ | -6.9 | 1.20E-19 | 33 | 20 | AEPRQEFEVMEDHAGTYGLGDRKDQGGYTMHQDQEGDTDAGLK...AEEAGIGDTPSLEDEAAGHVT + acA2 + oxM31/(M11) |
| 2-44...103-123 | 6905.0125 | 7+ | 3.7 | 6.20E-09 | 24 | 11 | AEPRQEFEVMEDHAGTYGLGDRKDQGGYTMHQDQEGDTDAGLK...AEEAGIGDTPSLEDEAAGHVT + acA2 + oxM11 + oxM31 |
| 2-44...103-124 | 7001.0813 | 6+ | 3.1 | 4.00E-28 | 42 | 25 | AEPRQEFEVMEDHAGTYGLGDRKDQGGYTMHQDQEGDTDAGLK...AEEAGIGDTPSLEDEAAGHVTQ + acA2 |
| 2-44...103-124 | 7017.0762 | 6+ | 2.6 | 1.40E-19 | 32 | 19 | AEPRQEFEVMEDHAGTYGLGDRKDQGGYTMHQDQEGDTDAGLK...AEEAGIGDTPSLEDEAAGHVTQ + acA2 + oxM31/(M11) |
| 2-44...103-124 | 7033.0711 | 6+ | 2.3 | 2.70E-18 | 32 | 19 | AEPRQEFEVMEDHAGTYGLGDRKDQGGYTMHQDQEGDTDAGLK...AEEAGIGDTPSLEDEAAGHVTQ + acA2 + oxM11 + oxM31 |
| 2-44...103-125 | 7072.1184 | 5+ | -6.5 | 1.80E-20 | 40 | 24 | AEPRQEFEVMEDHAGTYGLGDRKDQGGYTMHQDQEGDTDAGLK...AEEAGIGDTPSLEDEAAGHVTQA + acA2 |
| 2-44...103-125 | 7088.1133 | 6+ | 2.1 | 2.70E-19 | 34 | 25 | AEPRQEFEVMEDHAGTYGLGDRKDQGGYTMHQDQEGDTDAGLK...AEEAGIGDTPSLEDEAAGHVTQA + acA2 + oxM31/(M11) |
| 2-44...103-125 | 7104.1082 | 6+ | 2.9 | 5.70E-13 | 29 | 18 | AEPRQEFEVMEDHAGTYGLGDRKDQGGYTMHQDQEGDTDAGLK...AEEAGIGDTPSLEDEAAGHVTQA + acA2 + oxM11 + oxM31 |
| 2-44...103-126 | 7228.2195 | 6+ | 2.7 | 2.70E-03 | 11 | 15 | AEPRQEFEVMEDHAGTYGLGDRKDQGGYTMHQDQEGDTDAGLK...AEEAGIGDTPSLEDEAAGHVTQAR + acA2 |
| 2-44...103-126 | 7244.2144 | 6+ | -5.5 | 1.80E-04 | 3 | 10 | AEPRQEFEVMEDHAGTYGLGDRKDQGGYTMHQDQEGDTDAGLK...AEEAGIGDTPSLEDEAAGHVTQAR + acA2 + oxM31/(M11) |
| 2-44...103-127 | 7359.2600 | 6+ | 2.7 | 2.90E-05 | 11 | 12 | AEPRQEFEVMEDHAGTYGLGDRKDQGGYTMHQDQEGDTDAGLK...AEEAGIGDTPSLEDEAAGHVTQARM + acA2 |
| 2-44...103-127 | 7375.2549 | 7+ | 0.8 | 7.40E-09 | 16 | 13 | AEPRQEFEVMEDHAGTYGLGDRKDQGGYTMHQDQEGDTDAGLK...AEEAGIGDTPSLEDEAAGHVTQARM + acA2 + oxM69/(M31/M11) |
| 2-44...103-127 | 7407.2447 | 7+ | 5.6 | 3.80E-04 | 7 | 10 | AEPRQEFEVMEDHAGTYGLGDRKDQGGYTMHQDQEGDTDAGLK...AEEAGIGDTPSLEDEAAGHVTQARM + acA2 + oxM11 + oxM31 + oxM69 |
| 2-44...103-129 | 7545.3604 | 7+ | 1.7 | 1.60E-22 | 29 | 29 | AEPRQEFEVMEDHAGTYGLGDRKDQGGYTMHQDQEGDTDAGLK...AEEAGIGDTPSLEDEAAGHVTQARMVS + acA2 |
| 2-44...103-129 | 7561.3553 | 7+ | 2.9 | 4.70E-18 | 22 | 27 | AEPRQEFEVMEDHAGTYGLGDRKDQGGYTMHQDQEGDTDAGLK...AEEAGIGDTPSLEDEAAGHVTQARMVS + acA2 + oxM69/M31(/M11) |
| 2-44...103-129 | 7577.3503 | 7+ | 1.1 | 3.50E-12 | 22 | 17 | AEPRQEFEVMEDHAGTYGLGDRKDQGGYTMHQDQEGDTDAGLK...AEEAGIGDTPSLEDEAAGHVTQARMVS + acA2 + oxM69 + oxM31/(M11) |
| 2-44...103-129 | 7593.3452 | 7+ | -0.3 | 1.10E-08 | 19 | 16 | AEPRQEFEVMEDHAGTYGLGDRKDQGGYTMHQDQEGDTDAGLK...AEEAGIGDTPSLEDEAAGHVTQARMVS + acA2 + oxM11 + oxM31 + oxM69 |
| **1N** |  |  |  |  |  |  |  |
| 2-54 | 5920.5977 | 5+ | -5.6 | 1.60E-07 | 14 | 5 | AEPRQEFEVMEDHAGTYGLGDRKDQGGYTMHQDQEGDTDAGLKESPLQTPTED + acA2 |
| 2-65 | 6995.9919 | 5+ | -7.8 | 1.90E-03 | 10 | 2 | AEPRQEFEVMEDHAGTYGLGDRKDQGGYTMHQDQEGDTDAGLKESPLQTPTEDGSEEPGSETSD + acA2 |
| 2-65 | 7011.9868 | 6+ | 2.4 | 2.60E-03 | 11 | 4 | AEPRQEFEVMEDHAGTYGLGDRKDQGGYTMHQDQEGDTDAGLKESPLQTPTEDGSEEPGSETSD + acA2 + oxM31/(M11) |
| 2-65 | 7027.9817 | 6+ | 3.2 | 9.60E-03 | 8 | 5 | AEPRQEFEVMEDHAGTYGLGDRKDQGGYTMHQDQEGDTDAGLKESPLQTPTEDGSEEPGSETSD + acA2 + oxM11 + oxM31 |
| 2-66 | 7067.0290 | 6+ | 2.8 | 5.80E-04 | 11 | 4 | AEPRQEFEVMEDHAGTYGLGDRKDQGGYTMHQDQEGDTDAGLKESPLQTPTEDGSEEPGSETSDA + acA2 |
| 2-63...103-123 | 9831.2965 | 7+ | 1.0 | 2.30E-14 | 32 | 17 | AEPRQEFEVMEDHAGTYGLGDRKDQGGYTMHQDQEGDTDAGLKESPLQTPTEDGSEEPGSETSDAKSTPTAE...AEEAGIGDTPSLEDEAAGHVT + acA2 |
| 2-63...103-123 | 9847.2914 | 7+ | 3.2 | 3.60E-07 | 17 | 12 | AEPRQEFEVMEDHAGTYGLGDRKDQGGYTMHQDQEGDTDAGLKESPLQTPTEDGSEEPGSETSDAKSTPTAE...AEEAGIGDTPSLEDEAAGHVT + acA2 + oxM31/(M11) |
| 2-63...103-123 | 9863.2863 | 8+ | 1.6 | 4.10E-05 | 13 | 9 | AEPRQEFEVMEDHAGTYGLGDRKDQGGYTMHQDQEGDTDAGLKESPLQTPTEDGSEEPGSETSDAKSTPTAE...AEEAGIGDTPSLEDEAAGHVT + acA2 + oxM11 + oxM31 |
| 2-63...103-124 | 9959.3551 | 7+ | 3.9 | 7.60E-16 | 29 | 14 | AEPRQEFEVMEDHAGTYGLGDRKDQGGYTMHQDQEGDTDAGLKESPLQTPTEDGSEEPGSETSDAKSTPTAE...AEEAGIGDTPSLEDEAAGHVTQ + acA2 |
| 2-63...103-124 | 9975.3500 | 7+ | 1.8 | 3.10E-11 | 27 | 16 | AEPRQEFEVMEDHAGTYGLGDRKDQGGYTMHQDQEGDTDAGLKESPLQTPTEDGSEEPGSETSDAKSTPTAE...AEEAGIGDTPSLEDEAAGHVTQ + acA2 + oxM31/(M11) |
| 2-63...103-124 | 9991.3449 | 7+ | 1.5 | 3.80E-07 | 22 | 17 | AEPRQEFEVMEDHAGTYGLGDRKDQGGYTMHQDQEGDTDAGLKESPLQTPTEDGSEEPGSETSDAKSTPTAE...AEEAGIGDTPSLEDEAAGHVTQ + acA2 + oxM11 + oxM31 |
| 2-63...103-125 | 10030.3922 | 8+ | 1.4 | 8.00E-14 | 32 | 13 | AEPRQEFEVMEDHAGTYGLGDRKDQGGYTMHQDQEGDTDAGLKESPLQTPTEDGSEEPGSETSDAKSTPTAE...AEEAGIGDTPSLEDEAAGHVTQA + acA2 |
| 2-63...103-125 | 10046.3871 | 7+ | 1.9 | 1.10E-10 | 25 | 17 | AEPRQEFEVMEDHAGTYGLGDRKDQGGYTMHQDQEGDTDAGLKESPLQTPTEDGSEEPGSETSDAKSTPTAE...AEEAGIGDTPSLEDEAAGHVTQA + acA2 + oxM31/(M11) |
| 2-63...103-125 | 10062.3820 | 7+ | 2.8 | 1.20E-09 | 26 | 16 | AEPRQEFEVMEDHAGTYGLGDRKDQGGYTMHQDQEGDTDAGLKESPLQTPTEDGSEEPGSETSDAKSTPTAE...AEEAGIGDTPSLEDEAAGHVTQA + acA2 + oxM11 + oxM31 |
| 2-63...103-129 | 10503.6342 | 8+ | 5.7 | 3.70E-14 | 26 | 24 | AEPRQEFEVMEDHAGTYGLGDRKDQGGYTMHQDQEGDTDAGLKESPLQTPTEDGSEEPGSETSDAKSTPTAE...AEEAGIGDTPSLEDEAAGHVTQARMVS + acA2 |
| 2-63...103-129 | 10519.6291 | 8+ | 0.8 | 6.10E-07 | 19 | 21 | AEPRQEFEVMEDHAGTYGLGDRKDQGGYTMHQDQEGDTDAGLKESPLQTPTEDGSEEPGSETSDAKSTPTAE...AEEAGIGDTPSLEDEAAGHVTQARMVS + acA2 + oxM31/M98/(M11) |
| 2-63...103-129 | 10535.6241 | 7+ | 1.6 | 2.60E-04 | 15 | 14 | AEPRQEFEVMEDHAGTYGLGDRKDQGGYTMHQDQEGDTDAGLKESPLQTPTEDGSEEPGSETSDAKSTPTAE...AEEAGIGDTPSLEDEAAGHVTQARMVS + acA2 + oxM98/(M11) + M31 |
| **HT7** | **43 peptides** |  |  |  |  |  |  |
| **Peptide** | **Theoretical Mass [Da]** | **Charge** | **Δm [ppm]** | **expect** | **b-ions** | **y-ions** | **Sequence** |
| 130-173 | 4345.3214 | 5+ | -8.2 | 5.90E-02 | 1 | 6 | KSKDGTGSDDKKAKGADGKTKIATPRGAAPPGQKGQANATRIPA |
| 144-190 | 4596.4888 | 5+ | -7.0 | 8.20E-02 | 4 | 7 | GADGKTKIATPRGAAPPGQKGQANATRIPAKTPPAPKTPPSSGEPPK |
| 144-224 | 8031.1815 | 12+ | -10.7 | 2.10E-02 | 1 | 8 | GADGKTKIATPRGAAPPGQKGQANATRIPAKTPPAPKTPPSSGEPPKSGDRSGYSSPGSPGTPGSRSRTPSLPTPPTREPK |
| 145-190 | 4539.4673 | 5+ | -6.8 | 1.30E-02 | 3 | 7 | ADGKTKIATPRGAAPPGQKGQANATRIPAKTPPAPKTPPSSGEPPK |
| 151-189 | 3811.0493 | 6+ | 1.0 | 5.70E-05 | 11 | 2 | IATPRGAAPPGQKGQANATRIPAKTPPAPKTPPSSGEPP |
| 151-190 | 3939.1442 | 4+ | -4.8 | 2.90E-05 | 11 | 9 | IATPRGAAPPGQKGQANATRIPAKTPPAPKTPPSSGEPPK |
| 151-190 | 4019.1105 | 6+ | 3.0 | 4.60E-03 | 5 | 10 | ATPRGAAPPGQKGQANATRIPAKTPPAPKTPPSSGEPPK + pT181 |
| 153-189 | 3626.9281 | 5+ | 1.3 | 7.70E-04 | 11 | 1 | TPRGAAPPGQKGQANATRIPAKTPPAPKTPPSSGEPP |
| 153-190 | 3755.0231 | 5+ | -9.5 | 5.50E-04 | 8 | 8 | TPRGAAPPGQKGQANATRIPAKTPPAPKTPPSSGEPPK |
| 155-173 | 1860.0078 | 3+ | -6.1 | 9.70E-03 | 3 | 6 | RGAAPPGQKGQANATRIPA |
| 155-189 | 3428.8277 | 4+ | 0.9 | 8.70E-08 | 16 | 6 | RGAAPPGQKGQANATRIPAKTPPAPKTPPSSGEPP |
| 155-189 | 3508.7940 | 4+ | -6.4 | 5.70E-04 | 8 | 1 | RGAAPPGQKGQANATRIPAKTPPAPKTPPSSGEPP + pT181 |
| 155-190 | 3556.9226 | 4+ | -8.7 | 3.10E-12 | 13 | 10 | RGAAPPGQKGQANATRIPAKTPPAPKTPPSSGEPPK |
| 155-190 | 3636.8889 | 5+ | 0.2 | 2.70E-04 | 9 | 7 | RGAAPPGQKGQANATRIPAKTPPAPKTPPSSGEPPK + pT181 |
| 155-191 | 3643.9546 | 4+ | -6.5 | 8.20E-02 | 4 | 7 | RGAAPPGQKGQANATRIPAKTPPAPKTPPSSGEPPKS |
| 155-193 | 3816.0031 | 4+ | -7.6 | 7.50E-05 | 12 | 9 | RGAAPPGQKGQANATRIPAKTPPAPKTPPSSGEPPKSGD |
| 155-196 | 4116.1577 | 5+ | -8.1 | 4.40E-08 | 11 | 14 | RGAAPPGQKGQANATRIPAKTPPAPKTPPSSGEPPKSGDRSG |
| 155-224 | 6991.6153 | 10+ | -8.8 | 4.20E-02 | 3 | 7 | RGAAPPGQKGQANATRIPAKTPPAPKTPPSSGEPPKSGDRSGYSSPGSPGTPGSRSRTPSLPTPPTREPK |
| 155-224 | 7071.5817 | 9+ | -8.2 | 4.90E-02 | 7 | 5 | RGAAPPGQKGQANATRIPAKTPPAPKTPPSSGEPPKSGDRSGYSSPGSPGTPGSRSRTPSLPTPPTREPK + p |
| 156-173 | 1703.9067 | 3+ | 2.1 | 1.30E-08 | 4 | 11 | GAAPPGQKGQANATRIPA |
| 156-180 | 2423.3397 | 4+ | -10.3 | 1.80E-02 | 0 | 6 | GAAPPGQKGQANATRIPAKTPPAPK |
| 156-189 | 3272.7265 | 4+ | 0.3 | 1.00E-03 | 14 | 3 | GAAPPGQKGQANATRIPAKTPPAPKTPPSSGEPP |
| 156-189 | 3352.6929 | 4+ | 2.5 | 2.80E-02 | 11 | 2 | GAAPPGQKGQANATRIPAKTPPAPKTPPSSGEPP + p |
| 156-190 | 3400.8215 | 4+ | -6.4 | 4.80E-08 | 7 | 15 | GAAPPGQKGQANATRIPAKTPPAPKTPPSSGEPPK |
| 156-191 | 3487.8535 | 4+ | -6.6 | 2.50E-02 | 1 | 7 | GAAPPGQKGQANATRIPAKTPPAPKTPPSSGEPPKS |
| 156-193 | 3659.9019 | 5+ | 0.0 | 2.90E-06 | 4 | 12 | GAAPPGQKGQANATRIPAKTPPAPKTPPSSGEPPKSGD |
| 156-196 | 3960.0565 | 5+ | 0.5 | 6.80E-07 | 2 | 13 | GAAPPGQKGQANATRIPAKTPPAPKTPPSSGEPPKSGDRSG |
| 156-224 | 6835.5142 | 10+ | -10.3 | 4.70E-01 | 2 | 7 | GAAPPGQKGQANATRIPAKTPPAPKTPPSSGEPPKSGDRSGYSSPGSPGTPGSRSRTPSLPTPPTREPK |
| 157-189 | 3215.7051 | 3+ | 2.3 | 5.70E-03 | 0 | 7 | AAPPGQKGQANATRIPAKTPPAPKTPPSSGEPP |
| 157-190 | 3343.8000 | 5+ | 0.0 | 2.60E-08 | 3 | 19 | AAPPGQKGQANATRIPAKTPPAPKTPPSSGEPPK |
| 157-190 | 3423.7664 | 5+ | -0.4 | 7.20E-03 | 0 | 8 | AAPPGQKGQANATRIPAKTPPAPKTPPSSGEPPK + pT181 |
| 157-191 | 3430.8321 | 4+ | -8.4 | 2.50E-02 | 0 | 7 | AAPPGQKGQANATRIPAKTPPAPKTPPSSGEPPKS |
| 157-193 | 3602.8805 | 4+ | -6.5 | 1.60E-02 | 0 | 8 | AAPPGQKGQANATRIPAKTPPAPKTPPSSGEPPKSGD |
| 157-196 | 3903.0351 | 4+ | -6.4 | 6.00E-06 | 0 | 13 | AAPPGQKGQANATRIPAKTPPAPKTPPSSGEPPKSGDRSG |
| 157-224 | 6778.4928 | 9+ | -7.8 | 2.90E-04 | 0 | 11 | AAPPGQKGQANATRIPAKTPPAPKTPPSSGEPPKSGDRSGYSSPGSPGTPGSRSRTPSLPTPPTREPK |
| 158-173 | 1575.8481 | 3+ | -9.0 | 1.50E-05 | 0 | 7 | APPGQKGQANATRIPA |
| 158-189 | 3144.6680 | 4+ | 1.2 | 8.90E-10 | 19 | 4 | APPGQKGQANATRIPAKTPPAPKTPPSSGEPP |
| 158-189 | 3224.6343 | 4+ | 1.0 | 1.70E-02 | 11 | 4 | APPGQKGQANATRIPAKTPPAPKTPPSSGEPP + pT181 |
| 158-190 | 3272.7629 | 4+ | -8.3 | 1.70E-11 | 11 | 20 | APPGQKGQANATRIPAKTPPAPKTPPSSGEPPK |
| 158-191 | 3359.7950 | 4+ | -7.8 | 9.40E-03 | 4 | 10 | APPGQKGQANATRIPAKTPPAPKTPPSSGEPPKS |
| 158-193 | 3531.8434 | 4+ | 0.2 | 9.70E-06 | 5 | 10 | APPGQKGQANATRIPAKTPPAPKTPPSSGEPPKSGD |
| 158-196 | 3831.9980 | 5+ | 1.9 | 1.00E-06 | 6 | 13 | APPGQKGQANATRIPAKTPPAPKTPPSSGEPPKSGDRSG |
| 158-224 | 6707.4556 | 9+ | -9.1 | 8.30E-04 | 2 | 10 | APPGQKGQANATRIPAKTPPAPKTPPSSGEPPKSGDRSGYSSPGSPGTPGSRSRTPSLPTPPTREPK |
| **BT2** | **21 peptides** |  |  |  |  |  |  |
| **Peptide** | **Theoretical Mass [Da]** | **Charge** | **Δm [ppm]** | **expect** | **b-ions** | **y-ions** | **Sequence** |
| 151-224 | 7373.8369 | 10+ | -9.2 | 1.60E-02 | 2 | 8 | IATPRGAAPPGQKGQANATRIPAKTPPAPKTPPSSGEPPKSGDRSGYSSPGSPGTPGSRSRTPSLPTPPTREPK |
| 155-224 | 6991.6153 | 10+ | -0.2 | 1.40E-04 | 8 | 13 | RGAAPPGQKGQANATRIPAKTPPAPKTPPSSGEPPKSGDRSGYSSPGSPGTPGSRSRTPSLPTPPTREPK |
| 156-224 | 6835.5142 | 10+ | -9.0 | 9.00E-04 | 1 | 12 | GAAPPGQKGQANATRIPAKTPPAPKTPPSSGEPPKSGDRSGYSSPGSPGTPGSRSRTPSLPTPPTREPK |
| 157-224 | 6835.5142 | 10+ | -0.7 | 3.00E-03 | 0 | 10 | AAPPGQKGQANATRIPAKTPPAPKTPPSSGEPPKSGDRSGYSSPGSPGTPGSRSRTPSLPTPPTREPK |
| 161-224 | 6442.3130 | 10+ | -1.6 | 3.80E-02 | 1 | 9 | GQKGQANATRIPAKTPPAPKTPPSSGEPPKSGDRSGYSSPGSPGTPGSRSRTPSLPTPPTREPK |
| 168-224 | 5758.9779 | 7+ | -7.4 | 5.30E-03 | 6 | 6 | ATRIPAKTPPAPKTPPSSGEPPKSGDRSGYSSPGSPGTPGSRSRTPSLPTPPTREPK |
| 170-224 | 5586.8931 | 9+ | -9.3 | 2.60E-03 | 4 | 9 | RIPAKTPPAPKTPPSSGEPPKSGDRSGYSSPGSPGTPGSRSRTPSLPTPPTREPK |
| 172-210 | 3759.8452 | 5+ | 0.8 | 4.60E-03 | 6 | 9 | PAKTPPAPKTPPSSGEPPKSGDRSGYSSPGSPGTPGSRS |
| 173-224 | 5220.6552 | 8+ | 3.1 | 8.70E-04 | 3 | 13 | AKTPPAPKTPPSSGEPPKSGDRSGYSSPGSPGTPGSRSRTPSLPTPPTREPK |
| 174-223 | 5021.5231 | 7+ | 1.5 | 2.20E-02 | 2 | 9 | KTPPAPKTPPSSGEPPKSGDRSGYSSPGSPGTPGSRSRTPSLPTPPTREP |
| 174-224 | 5149.6181 | 7+ | -9.3 | 4.80E-02 | 1 | 8 | KTPPAPKTPPSSGEPPKSGDRSGYSSPGSPGTPGSRSRTPSLPTPPTREPK |
| 175-210 | 3463.6604 | 4+ | -4.6 | 1.30E-06 | 2 | 11 | TPPAPKTPPSSGEPPKSGDRSGYSSPGSPGTPGSRS |
| 175-220 | 4511.2317 | 5+ | -3.2 | 2.20E-03 | 3 | 10 | TPPAPKTPPSSGEPPKSGDRSGYSSPGSPGTPGSRSRTPSLPTPPT |
| 175-223 | 4893.4282 | 6+ | 0.3 | 3.60E-03 | 1 | 9 | TPPAPKTPPSSGEPPKSGDRSGYSSPGSPGTPGSRSRTPSLPTPPTREP |
| 175-224 | 5021.5231 | 7+ | 1.5 | 6.10E-07 | 2 | 15 | TPPAPKTPPSSGEPPKSGDRSGYSSPGSPGTPGSRSRTPSLPTPPTREPK |
| 180-220 | 4047.9886 | 5+ | 0.3 | 3.00E-03 | 1 | 9 | KTPPSSGEPPKSGDRSGYSSPGSPGTPGSRSRTPSLPTPPT |
| 180-224 | 4558.2800 | 6+ | 1.0 | 9.70E-04 | 1 | 11 | KTPPSSGEPPKSGDRSGYSSPGSPGTPGSRSRTPSLPTPPTREPK |
| 181-224 | 4430.1851 | 6+ | 0.6 | 6.60E-03 | 1 | 11 | TPPSSGEPPKSGDRSGYSSPGSPGTPGSRSRTPSLPTPPTREPK |
| 184-223 | 4006.9369 | 5+ | 2.1 | 4.90E-02 | 3 | 10 | SSGEPPKSGDRSGYSSPGSPGTPGSRSRTPSLPTPPTREP |
| 184-224 | 4135.0319 | 6+ | 0.3 | 5.10E-08 | 5 | 14 | SSGEPPKSGDRSGYSSPGSPGTPGSRSRTPSLPTPPTREPK |
| 192-224 | 3365.6712 | 4+ | -6.0 | 6.10E-03 | 6 | 8 | GDRSGYSSPGSPGTPGSRSRTPSLPTPPTREPK |
| **Tau_C123** | **23 peptides** |  |  |  |  |  |  |
| **Peptide** | **Theoretical Mass [Da]** | **Charge** | **Δm [ppm]** | **expect** | **b-ions** | **y-ions** | **Sequence** |
| **0N** |  |  |  |  |  |  |  |
| 2-44...103-123 | 6873.0227 | 6+ | -0.4 | 6.20E-18 | 32 | 16 | AEPRQEFEVMEDHAGTYGLGDRKDQGGYTMHQDQEGDTDAGLK...AEEAGIGDTPSLEDEAAGHVT + acA2 |
| 2-44...103-123 | 6889.0176 | 6+ | -1.0 | 3.10E-11 | 20 | 17 | AEPRQEFEVMEDHAGTYGLGDRKDQGGYTMHQDQEGDTDAGLK...AEEAGIGDTPSLEDEAAGHVT + acA2 + oxM31/M11 |
| 2-44...103-123 | 6905.0125 | 6+ | -2.2 | 5.90E-05 | 10 | 13 | AEPRQEFEVMEDHAGTYGLGDRKDQGGYTMHQDQEGDTDAGLK...AEEAGIGDTPSLEDEAAGHVT + acA2 + oxM11 + oxM31 |
| 9-44...103-123 | 5973.6090 | 6+ | 0.3 | 2.20E-05 | 13 | 10 | EVMEDHAGTYGLGDRKDQGGYTMHQDQEGDTDAGLK...AEEAGIGDTPSLEDEAAGHVT |
| 12-44...103-123 | 5614.4575 | 5+ | 0.6 | 3.50E-13 | 16 | 11 | EDHAGTYGLGDRKDQGGYTMHQDQEGDTDAGLK...AEEAGIGDTPSLEDEAAGHVT |
| 20-44...103-123 | 4784.1380 | 5+ | -0.5 | 3.90E-17 | 20 | 7 | LGDRKDQGGYTMHQDQEGDTDAGLK...AEEAGIGDTPSLEDEAAGHVT |
| 20-44...103-123 | 4800.1329 | 5+ | -0.3 | 1.80E-15 | 22 | 10 | LGDRKDQGGYTMHQDQEGDTDAGLK...AEEAGIGDTPSLEDEAAGHVT + oxM31 |
| 27-44...103-123 | 3971.7239 | 4+ | 0.5 | 8.10E-11 | 18 | 12 | GGYTMHQDQEGDTDAGLK...AEEAGIGDTPSLEDEAAGHVT |
| 31-44...103-123 | 3593.5700 | 4+ | 0.0 | 2.00E-15 | 21 | 13 | MHQDQEGDTDAGLK...AEEAGIGDTPSLEDEAAGHVT |
| 31-44...103-123 | 3609.5649 | 4+ | -1.0 | 4.10E-15 | 20 | 12 | MHQDQEGDTDAGLK...AEEAGIGDTPSLEDEAAGHVT + oxM31 |
| 32-44...103-123 | 3462.5295 | 4+ | 0.3 | 2.70E-13 | 17 | 12 | HQDQEGDTDAGLK...AEEAGIGDTPSLEDEAAGHVT |
| 34-44...103-123 | 3197.4120 | 3+ | -2.1 | 9.80E-12 | 13 | 16 | DQEGDTDAGLK...AEEAGIGDTPSLEDEAAGHVT |
| **1N** |  |  |  |  |  |  |  |
| 2-63...103-123 | 9831.2965 | 7+ | -0.1 | 8.80E-12 | 28 | 18 | AEPRQEFEVMEDHAGTYGLGDRKDQGGYTMHQDQEGDTDAGLKESPLQTPTEDGSEEPGSETSDAKSTPTAE...AEEAGIGDTPSLEDEAAGHVT + acA2 |
| 2-63...103-123 | 9847.2914 | 7+ | 0.7 | 1.30E-06 | 16 | 14 | AEPRQEFEVMEDHAGTYGLGDRKDQGGYTMHQDQEGDTDAGLKESPLQTPTEDGSEEPGSETSDAKSTPTAE...AEEAGIGDTPSLEDEAAGHVT + acA2 + oxM31/(M11) |
| 20-63...103-123 | 7742.4118 | 6+ | -1.7 | 3.20E-11 | 18 | 12 | LGDRKDQGGYTMHQDQEGDTDAGLKESPLQTPTEDGSEEPGSETSDAKSTPTAE...AEEAGIGDTPSLEDEAAGHVT |
| 21-63...103-123 | 7629.3277 | 6+ | -0.2 | 8.40E-06 | 9 | 14 | GDRKDQGGYTMHQDQEGDTDAGLKESPLQTPTEDGSEEPGSETSDAKSTPTAE...AEEAGIGDTPSLEDEAAGHVT |
| 31-63...103-123 | 6551.8438 | 5+ | -0.4 | 7.40E-11 | 20 | 14 | MHQDQEGDTDAGLKESPLQTPTEDGSEEPGSETSDAKSTPTAE...AEEAGIGDTPSLEDEAAGHVT |
| 31-63...103-123 | 6631.8101 | 5+ | -1.3 | 9.20E-08 | 9 | 14 | MHQDQEGDTDAGLKESPLQTPTEDGSEEPGSETSDAKSTPTAE...AEEAGIGDTPSLEDEAAGHVT + pS46 |
| 32-63...103-123 | 6420.8033 | 5+ | -2.5 | 3.20E-05 | 11 | 11 | HQDQEGDTDAGLKESPLQTPTEDGSEEPGSETSDAKSTPTAE...AEEAGIGDTPSLEDEAAGHVT |
| 44-63...103-123 | 5154.2920 | 4+ | -3.0 | 1.40E-06 | 8 | 14 | KESPLQTPTEDGSEEPGSETSDAKSTPTAE...AEEAGIGDTPSLEDEAAGHVT |
| 45-63...103-123 | 5026.1971 | 5+ | -0.4 | 2.90E-06 | 4 | 14 | ESPLQTPTEDGSEEPGSETSDAKSTPTAE...AEEAGIGDTPSLEDEAAGHVT |
| 50-63...103-123 | 4471.9270 | 4+ | 0.2 | 3.20E-09 | 13 | 14 | TPTEDGSEEPGSETSDAKSTPTAE...AEEAGIGDTPSLEDEAAGHVT |
| **2N** |  |  |  |  |  |  |  |
| 82-123 | 4138.9203 | 4+ | 0.9 | 4.00E-10 | 10 | 12 | EGAPGKQAAAQPHTEIPEGTTAEEAGIGDTPSLEDEAAGHVT |
| **Tau_C224** | **11 peptides** |  |  |  |  |  |  |
| **Peptide** | **Theoretical Mass [Da]** | **Charge** | **Δm [ppm]** | **expect** | **b-ions** | **y-ions** | **Sequence** |
| 184-224 | 4135.0319 | 5+ | -1.1 | 5.40E-01 | 0 | 5 | SSGEPPKSGDRSGYSSPGSPGTPGSRSRTPSLPTPPTREPK |
| 195-224 | 3037.5217 | 4+ | -3.4 | 5.90E-05 | 2 | 9 | SGYSSPGSPGTPGSRSRTPSLPTPPTREPK |
| 197-224 | 2893.4682 | 4+ | -2.4 | 8.50E-08 | 1 | 14 | YSSPGSPGTPGSRSRTPSLPTPPTREPK |
| 198-224 | 2730.4049 | 4+ | 0.9 | 9.00E-06 | 1 | 10 | SSPGSPGTPGSRSRTPSLPTPPTREPK |
| 199-224 | 2643.3729 | 4+ | -2.0 | 1.30E-03 | 4 | 9 | SPGSPGTPGSRSRTPSLPTPPTREPK |
| 200-224 | 2556.3409 | 4+ | -2.1 | 2.70E-01 | 1 | 7 | PGSPGTPGSRSRTPSLPTPPTREPK |
| 201-224 | 2459.2881 | 4+ | -3.6 | 1.50E-03 | 3 | 9 | GSPGTPGSRSRTPSLPTPPTREPK |
| 201-224 | 2539.2544 | 4+ | -2.6 | 9.50E-03 | 3 | 6 | GSPGTPGSRSRTPSLPTPPTREPK + p |
| 209-224 | 1819.0064 | 4+ | -2.7 | 6.50E-02 | 4 | 5 | RSRTPSLPTPPTREPK |
| 210-224 | 1662.9053 | 3+ | -2.6 | 5.80E-03 | 4 | 6 | SRTPSLPTPPTREPK |
| 214-224 | 1221.6717 | 3+ | -2.4 | 1.60E-02 | 1 | 6 | SLPTPPTREPK |

Supplementary tab. 4 Median and range (in brackets) of core biomarkers and fragments levels, age and gender of the AD and control groups in discovery (a) and validation (b) CSF cohorts.

|  |  | **T-tau  (pg/mL)** | **Aβ  (pg/mL)** | **P-tau  (pg/mL)** | **N-123  (pg/mL)** | **N-224 (pg/mL)** | **X-224 (pg/mL)** | **Age** | **Gender (n)** |
| --- | --- | --- | --- | --- | --- | --- | --- | --- | --- |
| a) | **AD**  (clinical diagnosis, AD biomarkers + | 807.5  (510-1130) | 427.5 (230-500) | 98  (70-151) | 456  (93-1480) | 52.7  (19-319) | - | 79.5 | M=10; F=10 |
|  | **Controls** (minor symptoms, basic & AD biomarkers -) | 210  (98-300) | 898  (658-1130) | 29.5  (20-48) | 89  (4-784) | 8.1  (3-25) | - | 67 | M=8; F=12 |
| b) | **AD**  (clinical diagnosis + IWG-2 biomarker criteria) | 849.8  (288-1773.4) | 238.4  (89.9-373.5) | 73.8  (29.2-135.5) | 426.3  (124.9-9341.6) | 27.4  (6-112.1) | 207.5  (42.5-473.5) | 74.5 | M=15; F=31 |
|  | **Controls**  (healthy elderly volunteers) | 435.6  (112.3-1156) | 532.7  (131.8-1030) | 47.7  (19.2-142.8) | 215.5  (21.4-12724.6) | 15.5  (4.4-94.3) | 134.6  (40.5-382.6) | 74.5 | M=16; F=34 |

Supplementary tab. 5 Median and range (in brackets) of core biomarkers and fragments levels, age and gender of the groups in the longitudinal CSF cohort.

|  | **t-tau**  **(pg/mL)** | **Aβ**  **(pg/mL)** | **p-tau**  **(pg/mL)** | **N-123**  **(pg/mL)** | **N-224**  **(pg/mL)** | **Age** | **Gender (n)** |
| --- | --- | --- | --- | --- | --- | --- | --- |
| **AD** | 568.5 | 418.5 | 83.5 | 43.8 | 112.1 | 70 | F=9 |
|  | (248-1064) | (218-863) | (27-132) | (18-140.2) | (31.2-325) |  | M=7 |
| **MCI** | 332 | 882 | 62.5 | 29.6 | 75.6 | 70 | F=17 |
|  | (125-1149) | (230-2066) | (29-136) | (4-264) | (23.6-385.4) |  | M=21 |
| **MCI-AD** | 623 | 472 | 87 | 48.2 | 158.6 | 71.5 | F=10 |
|  | (215-1215) | (231-745) | (50-300) | (16.5-211) | (21.1-795.5) |  | M=10 |
| **OND** | 174 | 857 | 40 | 15.6 | 43.9 | 63 | F=8 |
|  | (74-1192) | (360-1668) | (15-116) | (3.2-533.3) | (10.5-247) |  | M=13 |

Supplementary tab. 6 Median and range (in brackets) of core biomarkers and N-224 levels, mean age and gender of the groups in the PSP and CBS cohort. a) only amyloid negative subjects; b) amyloid negative and amyloid positive subjects.

|  |  | **T-tau  (pg/mL)** | **Aβ  (pg/mL)** | **P-tau  (pg/mL)** | **N-224 (pg/mL)** | **Age at onset** | **Gender (n)** |
| --- | --- | --- | --- | --- | --- | --- | --- |
| a) | **PSP (only Aβ-)** | 277.2  (182.8-494.6) | 780  (573-958) | 38  (23-182) | 2.5  (0.1-3.6) | 66.8 | M=7; F=9 |
|  | **CBS (only Aβ-)** | 292.5  (193.1-529.5) | 752  (590-1040) | 34  (28-52) | 5.2  (0.3-14.8) | 59.9 | M=2; F=5 |
| b) | **PSP (Aβ+ and Aβ-)** | 285.2  (126.7-517.2) | 540  (190-958) | 35  (19-182) | 2.6  (0.1-13.7) | 65 | M=14; F=18 |
|  | **CBS (Aβ+ and Aβ-)** | 292.5  (107.1-918.8) | 450  (160-1040) | 36  (16-83) | 4.1  (0.3-15.2) | 64 | M=5; F=10 |

Supplementary tab. 7 Mean age, N-224, MMSE and ADAS-Cog scores, gender and treatment in the biomarker stability study CSF cohort. In brackets, range of N-224, MMSE and ADAS-Cog score.

|  | **Age** | **Gender (n)** | **Treatment** |
| --- | --- | --- | --- |
|  | 75.7 | M=26; F=25 | Donepezil (22)  Galantamine (22)  Rivastigmine (7) |
|  | **MMSE** | **ADAS-Cog** | **N-224 (pg/mL)** |
| Baseline | 24.2 (16-30) | 14.3 (1.3-31.7) | 79 (5-566) |
| Follow-up | 22.9 (15-30) | 14.2 (1.0–30.3) | 79 (7-506) |

Assay validation

Method

For assay validation, several plates were run at different occasions. Calibrators and low and high quality control (QC) samples were included in duplicate in the ELISA assays (N-123, x-224), while in the N-224 assay calibrators were run in triplicate. LLOQ and ULOQ were determined by analyzing the deviation from the true value of each calibrator point. LOD was determined by analyzing 16 duplicates of the blank and by adding 3 standard deviations to the mean blank signal (16 replicates/one plate). Precision is defined by the calculated standard deviation (SDr) and the variation coefficient (CVr) using One-way ANOVA in accordance with ISO 5257-2. Neo-epitope specific antibodies anti-tau 123 and anti-tau 224 were tested for cross-reactivity using direct ELISA. Each clone was tested against full-length 2N4R Tau, 123- and 224-fragments. Tau 12 was used as positive control.

Results

In the N-123 assay (Fig. 1b), LLOQ and ULOQ were respectively 12.2 pg/mL and 12500 pg/mL, determined as described above. The CV% for the back calculated concentrations of the data from the calibrator curve was <20% at LLOQ and ULOQ. Within- and between-plate variability were measured over 3 runs (11 plates) and were, respectively, 7.2% and 18.6% for the high QC sample and 3.8% and 26.1% for the low QC sample. LOD was 10.3 pg/mL. Samples under LLOQ were four in the discovery cohort, three in the validation cohort, three in the longitudinal cohort, eight in the CBS cohort and 19 in the PSP cohort.
In the N-224 assay (Fig.1c), LLOQ and ULOQ were respectively 2.5 pg/mL and 160 pg/mL. The CV% for the back calculated concentrations of the data from the calibrator curve was <20% at LLOQ and ULOQ. The LOD was 0.05 pg/mL. In the discovery cohort, within-run variability was 2.7 % for the low QC and 4.8 % for the high QC. In the validation cohort, within-run variability was 3.6 % for the low QC and 1.5 % for the high QC. QC samples were run in two duplicates in each study. Samples under LLOQ were two in the discovery cohort and one in the longitudinal cohort.
In the x-224 assay (Fig. 1c), LLOQ and ULOQ were respectively 15.6 pg/mL and 2000 pg/mL. The CV% for the back calculated concentrations of the data from the calibrator curve was <20% at LLOQ and ULOQ. Within- and between-plate variability were measured over 9 runs (15 plates) and were, respectively, 11.4% and 20.7% for the high QC sample and 14.8% and 34.3% for the low QC sample. LOD was 0.03 pg/mL.
No cross-reactivity was found in neither of the neo-epitope specific antibodies against full-length tau or the non-specific fragment. Tau 12 bound to all three tau antigens.
